# A VEGF-dependent gene signature enriched in mesenchymal ovarian cancer predicts patient prognosis

**DOI:** 10.1038/srep31079

**Published:** 2016-08-08

**Authors:** Xia Yin, Xiaojie Wang, Boqiang Shen, Ying Jing, Qing Li, Mei-Chun Cai, Zhuowei Gu, Qi Yang, Zhenfeng Zhang, Jin Liu, Hongxia Li, Wen Di, Guanglei Zhuang

**Affiliations:** 1 State Key Laboratory of Oncogenes and Related Genes, Renji-Med X Clinical Stem Cell Research Center, Ren Ji Hospital, School of Medicine, Shanghai Jiao Tong University, Shanghai, China; 2 Department of Obstetrics and Gynecology, Ren Ji Hospital, School of Medicine, Shanghai Jiao Tong University, Shanghai, China; 3 Shanghai Key Laboratory of Gynecologic Oncology, Ren Ji Hospital, School of Medicine, Shanghai Jiao Tong University, Shanghai, China; 4 Department of Obstetrics and Gynecology, Shanghai General Hospital, Shanghai Jiao Tong University School of Medicine, Shanghai, China; 5 Department of Obstetrics and Gynecology, Beijing Shijitan Hospital, Capital Medical University, Beijing, China; 6 State Key Laboratory of Oncogenes and Related Genes, Shanghai Cancer Institute, Ren Ji Hospital, School of Medicine, Shanghai Jiao Tong University, Shanghai, China; 7 Lingyun Community Health Service Center of Xuhui District, Shanghai, China

## Abstract

We have previously reported surrogate biomarkers of VEGF pathway activities with the potential to provide predictive information for anti-VEGF therapies. The aim of this study was to systematically evaluate a new VEGF-dependent gene signature (VDGs) in relation to molecular subtypes of ovarian cancer and patient prognosis. Using microarray profiling and cross-species analysis, we identified 140-gene mouse VDGs and corresponding 139-gene human VDGs, which displayed enrichment of vasculature and basement membrane genes. In patients who received bevacizumab therapy and showed partial response, the expressions of VDGs (summarized to yield VDGs scores) were markedly decreased in post-treatment biopsies compared with pre-treatment baselines. In contrast, VDGs scores were not significantly altered following bevacizumab treatment in patients with stable or progressive disease. Analysis of VDGs in ovarian cancer showed that VDGs as a prognostic signature was able to predict patient outcome. Correlation estimation of VDGs scores and molecular features revealed that VDGs was overrepresented in mesenchymal subtype and BRCA mutation carriers. These findings highlighted the prognostic role of VEGF-mediated angiogenesis in ovarian cancer, and proposed a VEGF-dependent gene signature as a molecular basis for developing novel diagnostic strategies to aid patient selection for VEGF-targeted agents.

High-grade serous ovarian carcinoma (HGS-OvCa) is the predominant form of ovarian cancer and the most lethal gynecological malignancy, with approximately 140,000 deaths per year globally^1^,^2^,^3^. The majority of patients are diagnosed as advanced, disseminated disease and the survival rate is dismal^4^,^5^. In addition, recent analyses from The Cancer Genome Atlas (TCGA) research network have identified four molecular subtypes of HGS-OvCa, namely Differentiated, Immunoreactive, Mesenchymal and Proliferative, indicating that a high level of intertumoral heterogeneity may also impact on patient outcome^6^. As a result, despite our increased understanding of the physiopathology underpinning HGS-OvCa, its clinical management has not been appreciably improved over the past decades^7^. The current standard treatment of HGS-OvCa is aggressive surgical debulking followed by multi-cycles of platinum-based combination chemotherapy. Although many patients display a transient response, the vast majority eventually relapse and suffer from recurrent disease without efficacious treatment regimen^8^,^9^. Therefore, there is a compelling need to develop novel therapeutic strategies that can effectively control advanced-stage HGS-OvCa^10^,^11^.

VEGF-mediated tumor angiogenesis has been prominently implicated in the progression of ovarian cancer and hence represents one of the most promising targets^12^,^13^,^14^,^15^. Triggered by the remarkable preclinical efficacy of bevacizumab (Avastin), a humanized VEGF blocking monoclonal antibody, in a range of solid tumor types, a series of clinical studies have been conducted to evaluate bevacizumab in patients with newly-diagnosed or recurrent ovarian cancer. Two large prospective randomized phase III trials (GOG-0218 and ICON7) met their primary objective, demonstrating significantly improved progression-free survival (PFS) with bevacizumab administered with front-line chemotherapy compared with chemotherapy alone^16^,^17^. Two further randomized phase III clinical trials (OCEANS and AURELIA) have proved the efficacy of bevacizumab in recurrent ovarian cancer^18^,^19^. Based on these results, bevacizumab received European and FDA regulatory approval for use in combination with chemotherapy to treat advanced-stage ovarian cancer. Nevertheless, the increased PFS did not translate into a significant improvement in overall survival (OS) and robust biomarkers for predicting bevacizumab efficacy are currently lacking, which impedes patient selection and the optimal use of bevacizumab in ovarian cancer^20^,^21^.

We have previously employed gene expression profiling analysis and identified surrogate markers of VEGF inhibition as potential guides for the selection of patients who likely benefit from anti-VEGF therapy. The selected gene set was able to inform on VEGF downstream bioactivity and predict clinical outcome in breast cancer following bevacizumab treatment^22^. In this study, through further characterization of angiogenesis-related gene transcripts in mouse models and human samples, we established a novel VEGF-dependent gene signature and investigated its correlation with molecular subtypes of ovarian cancer and patient prognosis.

## Results

### Identification of a VEGF-dependent gene signature

**Figure 1:**
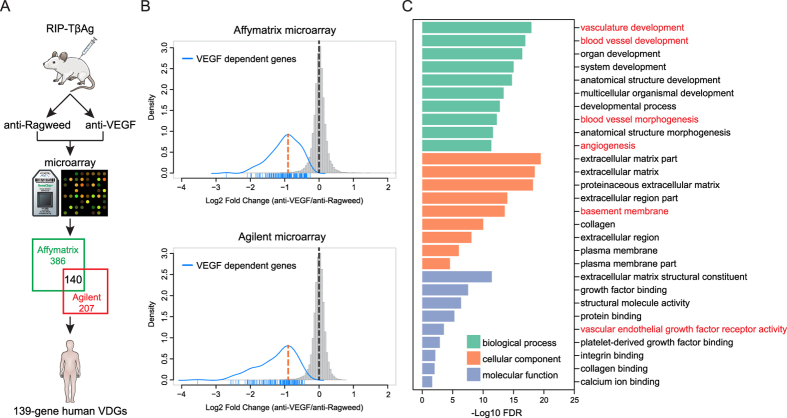
Identification of a VEGF-dependent gene signature. (**A**) A schematic overview of the study. (**B**) Density plots from microarray analysis of anti-VEGF vs. anti-Ragweed treated tumors. Expression levels of VDGs (shown as blue lines) decreased significantly relative to all genes (gray histogram). The dashed red line indicates the mean fold change for VDGs. The dashed black line indicates the mean change for all the genes. (**C**) Gene ontology categories overrepresented in VDGs. The terms directly related to vasculature were highlighted in red.

In order to generate a faithful VEGF-dependent gene signature, we systematically profiled the transcriptional changes induced by VEGF neutralization in a transgenic murine model of highly vascularized pancreatic neuroendocrine tumors, using two well-established microarray platforms in two independent experiments (Fig. 1A). As we reported previously^22^, anti-VEGF treatment displayed merely anti-vascular but not anti-proliferative effects at day 7, which was selected as the time point to characterize the specific gene expression response of the tumor vasculature due to VEGF blockade. Affymatrix and Agilent microarray analysis identified 386 and 207 genes with a significant decrease (adjusted *P* value < 0.05) in transcript abundance, respectively (Supplementary Tables 1 and 2). We focused on the 140 genes detected by both platforms to further minimize false-positives associated with genome-wide profiling assays (Supplementary Table 3), and termed these genes VDGs (VEGF-dependent gene signature). Notably, we observed no corresponding upregulation of gene expression with one only exception Oxct1, consistent with the physical elimination of tumor vascular endothelial cells (Fig. 1B). Functional annotation demonstrated that the VDGs was enriched for endothelial or basement membrane specific genes (such as Cdh5 and Collagens), and was implicated in blood vessel morphogenesis (Fig. 1C; Supplementary Table 4). In addition, the VDGs was composed of both candidate proximal biomarkers of VEGF inhibition (e.g. Esm1, Nid2 and Prnd) and more distal downstream surrogate markers of the subsequent vessel loss (e.g. Cdh5, Plvap and Flt1), as we discovered previously^22^.

### Validation of the VEGF-dependent gene signature

**Figure 2:**
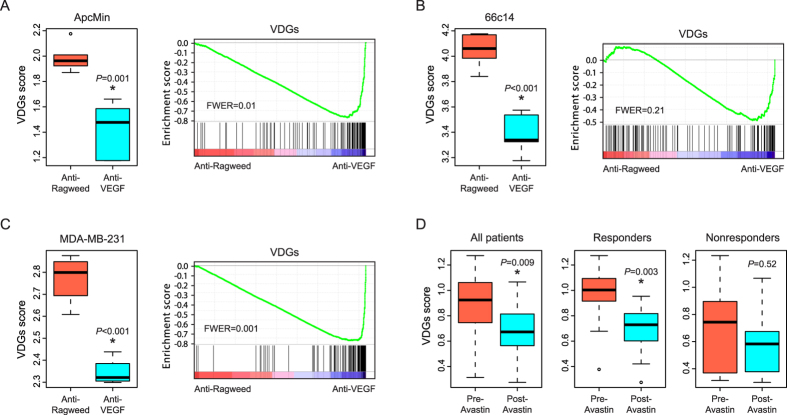
Validation of the VEGF-dependent gene signature. (**A**) Downregulation of the VDGs following anti-VEGF treatment in the ApcMin genetic tumor model. (**B**) Downregulation of the VDGs following anti-VEGF treatment in an orthotopic 66c14 mouse breast cancer model. (**C**) Downregulation of the VDGs following anti-VEGF treatment in a subcutaneous human breast carcinoma MDA-MB-231 model. (**D**) Changes of the VDGs scores in serial clinical specimens collected from breast cancer patients treated with neoadjuvant bevacizumab.

Next, we sought to validate the VDGs as an indicator of VEGF signaling activity in various tumor models. The expressions of VDGs were summarized to yield VDGs scores by using single sample gene set enrichment analysis. Firstly, we analyzed transcriptional responses to long-term anti-VEGF treatment in samples from a murine genetic intestinal tumor model ApcMin^23^. As expected, anti-VEGF administration was sufficient to induce a significant decrease in the mean value of VDGs scores (Fig. 2A). Secondly, in an orthotopic 66c14 mouse breast cancer model, the VDGs was similarly downregulated by short-term anti-VEGF treatment (Fig. 2B). Thirdly, anti-VEGF exposure resulted in a statistically significant reduction of VDGs scores in an established subcutaneous human breast carcinoma tumor model (MDA-MB-231) (Fig. 2C). Therefore, regardless of the different tumor models and independent of the length of anti-VEGF treatment, the VDGs consistently reflected the anti-vascular consequences of VEGF signaling inhibition and likely correlated with the VEGF pathway bioactivity in tumor samples.

Importantly, we assessed the dynamic changes of VDGs scores in serial clinical specimens collected from breast cancer patients treated with neoadjuvant bevacizumab. In this cohort, twenty treatment-naïve patients were biopsied and received one cycle of bevacizumab followed by six cycles of bevacizumab plus combination chemotherapy prior to surgery^24^. The 139 human orthologs of the VDGs exhibited a significant downregulation in post-bevacizumab tissues compared with matched pretreatment biopsies (Fig. 2D). Of more interest, when the patients were stratified into two subgroups based on the Response Evaluation Criteria in Solid Tumors (RECIST), VDGs scores were markedly decreased by bevacizumab treatment specifically in responders (partial response), but were not significantly altered in nonresponders (stable or progressive disease) (Fig. 2D; Supplementary Figure 1). Additionally, we observed a trend towards higher baseline VDGs scores in responders than nonresponders (Supplementary Figure 2), suggesting that elevated VEGF pathway bioactivity might be associated with the clinical benefit from bevacizumab therapy. Taken together, the new VDGs that we identified in preclinical models enables the accurate detection of an evolutionary conserved vascular response to VEGF signaling inhibition in clinical tumor samples.

### The VDGs predicts patient prognosis in HGS-OvCa

**Figure 3:**
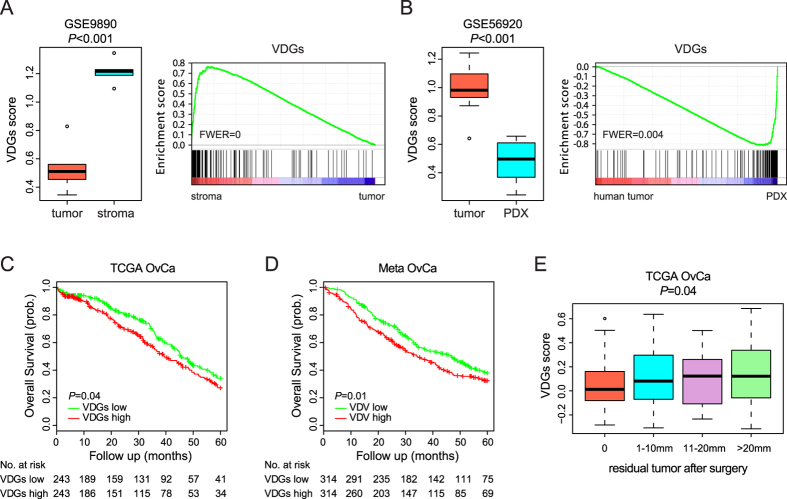
The VDGs predicts patient prognosis in HGS-OvCa. (**A**) Upregulation of the VDGs in microdissected tumor stroma versus epithelial tissues (5 samples). (**B**) Downregulation of the VDGs in PDX versus matched primary tumors (9 samples). (**C**) Kaplan Meier curves for the two prognostic groups of TCGA samples classified by the VDGs. (**D**) Kaplan Meier curves for meta-analysis of 681 HGS-OvCa expression profiles across four cohorts. (**E**) Higher VDGs scores in HGS-OvCa patients with residual disease after debulking surgery.

Although bevacizumab has been approved for treating advanced-stage ovarian cancer, predictive biomarkers to improve patient selection are needed. Considering the tempting hypothesis that higher levels of VEGF downstream bioactivity potentially correlate with increased responses to bevacizumab, we reasoned that it might be important to comprehensively characterize the VDGs in the context of ovarian cancer. To this end, we investigated the VDGs in a gene set of ovarian tumor samples, in which epithelial and stromal components had been microdissected and profiled separately. Both ssGSEA and GSEA indicated that the VDGs was significantly enriched in the microdissected stroma components in comparison to paired tumor cells (Fig. 3A). In addition, we analyzed gene expression profiles of nine pairs of ovarian tumors and matched patient-derived xenografts (PDXs), in which mouse cells should competitively substitute human stroma including endothelial cells. The VDGs scores were accordingly decreased in PDXs (Fig. 3B). Our analyses also indicated that the VDGs scores increased upon chemotherapy or tumor metastasis (Supplementary Figure 3), although the clinical significance of these findings was not yet clear.

To assess the VDGs as a prognostic biomarker, we segregated the 486 TCGA HGS-OvCa samples into two clusters, i.e. the ‘VDGs low’ group and the ‘VDGs high’ group, according to the median VDGs scores. We found that patients classified as ‘VDGs high’ had significantly shorter survival than ‘VDGs low’ patients (hazard ratio: 1.30; 95% CI: 1.01 to 1.68) (Fig. 3C). To independently corroborate the prognostic value of VDGs, we performed meta-analysis of 681 HGS-OvCa expression profiles across four large published datasets. Each of the four HGS-OvCa cohorts displayed a trend for decreased overall survival in the ‘VDGs high’ group versus ‘VDGs low’ group but did not achieve statistical significance (Supplementary Figure 4). However, when we combined all four datasets together, the difference in overall survival between the two groups was statistically significant (hazard ratio: 1.30; 95% CI: 1.06 to 1.60) (Fig. 3D). Interestingly, the VDGs did not predict the responsiveness to chemotherapy (Supplementary Figure 5), but was found to correlate with residual disease after debulking surgery (Fig. 3E), suggesting that it could be a potential molecular biomarker for accurate identification of patients at high risk of suboptimal cytoreduction. We conclude that the VDGs is of prognostic value in patients with HGS-OvCa.

### The VDGs is enriched in mesenchymal ovarian tumors

**Figure 4:**
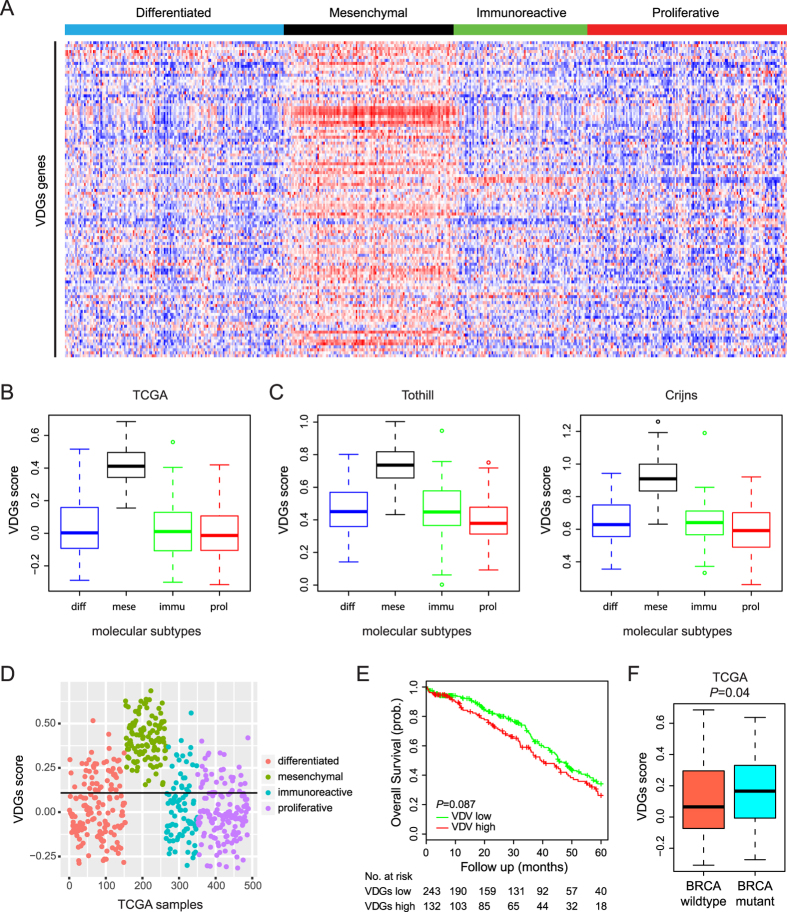
The VDGs is enriched in mesenchymal ovarian tumors. (**A**) Heatmap of the VDGs gene expression for four molecular subtypes of TCGA samples. Red color, high expresion; blue color, low expression. (**B**) Summarized VDGs scores in four molecular subtypes of TCGA samples. (**C**) Summarized VDGs scores in four molecular subtypes of Tothill and Crijns cohorts. (**D**) VDGs scores of individual samples in the TCGA dataset. The black line marks the median VDGs score of all samples. (**E**) Kaplan Meier curves for the two prognostic groups of TCGA samples excluding mesenchymal ovarian tumors. (**F**) Upregulation of the VDGs in ovarian cancer with BRCA1/BRCA2 mutations.

Previous studies have identified four HGS-OvCa subtypes, namely Differentiated, Mesenchymal, Immunoreactive and Proliferative, which exhibit distinct transcriptional, biological and clinical characteristics^6^,^25^. We speculated that these molecular subtypes might have differential expression of VDGs and accordingly differ in their response to anti-VEGF agents. Therefore, we explored the potential differences in transcriptional levels of the 139 VDGs genes among the four TCGA subtypes. Indeed, the vast majority of VDGs genes appeared to be relatively highly expressed in the Mesenchymal cluster (Fig. 4A), in which the summarized VDGs scores were significantly increased (Fig. 4B). Similar results were obtained with Tothill and Crijns cohorts (Fig. 4C), strongly indicating that the VDGs was enriched in mesenchymal ovarian tumors. When we stratified the TCGA specimens into two groups based on the median VDGs scores, 100% (111/111) of the Mesenchymal tumors were classified as ‘VDGs high’, in comparison to 40% (61/153), 33% (28/85) and 31% (43/137) of the Differentiated, Immunoreactive and Proliferative samples, respectively (Fig. 4D). Of note, even in non-mesenchymal tumors, higher VDGs scores still tended to predict poorer patient outcome (Fig. 4E). Lastly, the BRCA1/BRCA2 status was available for 316 TCGA patients (Supplementary Table 5) and the VDGs was overrepresented in BRCA mutation carriers (Fig. 4F).

## Discussion

In this study, we extended previous work of exploiting gene expression profiling approach for accurate detection of VEGF downstream biological activity, and designed a more stringent analytical protocol to identify and validate a reliable VEGF-dependent gene signature in preclinical tumor models and in human patients. Although the VDGs overlapped with previously developed VEGF-related signatures in general^22^,^26^, the refined gene list presented here was physiologically relevant on the basis of suitable *in vivo* models and integration of gene profiling results from two independent microarray platforms. Furthermore, we presented a detailed analysis of the VDGs in ovarian cancer by systematically interrogating TCGA HGS-OvCa expression data and four additional genome-wide transcriptome cohorts in the public domain. As the VDGs directly reflected vascular-specific anti-VEGF downstream effects in various experimental models and clinical dataset, an exquisite delineation of its relation to molecular subtypes and disease prognosis in ovarian cancer may advance our understanding of the VEGF pathway and provide a promising diagnostic strategy for identifying a patient subpopulation likely to derive benefit from anti-VEGF treatment. Further optimization and validation of the VDGs in both retrospective and prospective clinical studies will pave the way to developing a biomarker-based companion assay for predicting responsiveness to bevacizumab in the clinical management of ovarian cancer patients.

Tumor angiogenesis is characterized by extensive molecular regulation, aberrant endothelial morphology, constant vessel remodeling and loss of hierarchical architecture^27^,^28^. The complexity and heterogeneity of tumor vasculature not only make pathological assessments of microvascular dependency challenging, but also impede biomarker discovery for pharmacological perturbations of tumor endothelium. As a result, overall microvascular density has variable prognostic capacity in predicting outcome of cancer patients^29^,^30^. Similarly, neither regulators of endothelial proliferation nor expression of vascular specific genes consistently correlate with bevacizumab efficacy across indications^31^,^32^. Our results, together with other reports^22^, collectively suggest that transcriptome-level characterization of the distinct tumor vascular compartment directly responsive to VEGF signaling inhibition may better inform on the biological activity and functional significance of VEGF-mediated tumor angiogenesis. This drug-gene signature-disease connection is reminiscent of the “Connectivity Map” concept^33^,^34^, which assists to elucidate the complex mechanisms underlying a biological pathway or pharmacological modulation. Hence, the system biology approach may be extremely powerful in understanding the intricate tumor microvasculature.

Ovarian cancer is a heterogeneous disease and its predominant form, HGS-OvCa, contains at least four molecular subtypes, i.e. Differentiated, Mesenchymal, Immunoreactive and Proliferative. We have previously provided evidence that tumor-infiltrating stromal cells had a profound effect on the expression patterns of HGS-OvCa as well as patient prognosis^35^. Endothelial cells are considered as important constituents of cancer-supporting stroma, and the VDGs is indeed enriched in the noncancerous stromal components of ovarian tumors. Therefore, tumor vasculature, in conjunction with other stromal composition, may also contribute to defining molecular subtypes of HGS-OvCa. Alternatively, the VDGs and VEGF-regulated endothelium could independently mark a specific HGS-OvCa subpopulation with unique pathophysiological properties. Although a simple stratification of HGS-OvCa cases based on median VDGs scores is proved to be prognostically predictive, prospectively refined gene sets and tailored threshold may further improve the performance of this signature classifier. In addition, previous studies have identified multiple prognostic signatures in ovarian cancer^36^,^37^,^38^,^39^,^40^,^41^, and it would be interesting to determine whether the VDGs led to the same or different patient stratification in comparison to published models.

Our observation that the VDGs is overrepresented in the Mesenchymal subtype is particularly intriguing. Mechanistically, the intimate interplay between endothelial cells and fibroblasts/pericytes may promote vigorous angiogenic sprouting in mesenchymal tumors. Conceivably, the Mesenchymal subtype of ovarian cancer would derive increased benefit from anti-angiogenic therapy. Supporting this hypothesis, a preliminary analysis of response to bevacizumab in relation to the molecular classifications has indicated an improvement of PFS for patients in the Mesenchymal subtype of ovarian cancer^42^. It is noteworthy that a similar distinct subtype with mesenchymal features has been identified in multiple other tumor types including colon cancer, breast cancer and glioblastoma^43^,^44^,^45^,^46^,^47^. Therefore, it would be interesting to determine whether the VDGs is enriched in mesenchymal tumors and whether the enrichment translates to anti-VEGF efficacy across different cancers. Along this line, recent retrospective analysis of AVAglio data showed that both mesenchymal and proneural tumors derived a PFS benefit from bevacizumab compared with placebo in glioblastoma^48^.

In conclusion, we proposed a VEGF-dependent gene signature as a molecular basis for developing novel diagnostic and therapeutic strategies. Our data indicated the prognostic value of VDGs in ovarian cancer. The VDGs enrichment in ovarian cancer with mesenchymal signatures or BRCA mutations suggested that these patients might most likely gain sustained benefit from bevacizumab therapy, which requires further investigation in retrospective and prospective studies.

## Methods

### Mouse models and treatment regimens

RIP-TβAg mice, ApcMin mice and Beige Nude mice were housed and cared for according to guidelines from the Institutional Animal Care and Use Committee (IACUC) of Renji Hospital, and all the animal experiments were approved by Renji Hospital IACUC. B20-4.1.1 (anti-VEGFA) and anti-Ragweed were dosed by intraperitoneal injection at 5 mg/kg twice weekly as described previously^22^.

### Microarray analysis and gene signature derivation

Tumor RNA was prepared with RNeasy Plus Mini Kit (Qiagen) according to the manufacturer’s protocol. Total RNA was subjected to microarray analysis with Affymetrix Mouse Genome 430 2.0 Array or Agilent Whole Mouse Genome Oligo Microarray. Five biological replicates per treatment group were included for statistical analyses. All microarray analyses were performed using the R Statistical language. Affymetrix microarray probe-level data were normalized and summarized using robust multiarray average procedure (RMA)^49^. Agilent data were lowess normalized and log transformed, and the mean was used to calculate gene level summaries. Differential gene expression was analyzed with linear models for microarray data (Limma)^50^. The VEGF-dependent gene signature was derived from genes significantly (adjusted *P* value < 0.05) downregulated in anti-VEGF treated tumors compared to controls.

### HGS-OvCa microarray datasets

The microarray datasets of HGS-OvCa used in current study are publicly available and as described in our previous report^35^. Combined and filter TCGA gene expression data were downloaded from https://tcga-data.nci.nih.gov/docs/publications/ov_2011/. The BRCA mutation status of TCGA samples was based on http://www.cbioportal.org/. The four patient cohorts (Tothill, Crijns, Bonome, and Yoshihara) for meta-analysis have been described previously^51^,^52^,^53^,^54^, and processed data were downloaded from a recent paper^37^. Other microarray datasets, including GSE9890, GSE15622, GSE30587 and GSE56920^51^,^55^,^56^,^57^, were obtained from the Gene Expression Omnibus.

### Molecular subtypes of ovarian tumors

Molecular subtypes of ovarian tumors were identified as previously described^35^. Briefly, TCGA HGS-OvCa samples were classified based on non-negative matrix factorization (NMF) consensus clustering. To minimize the impact of outlier samples on the identification of subtype markers, the silhouette width was computed to filter out expression profiles with negative values. Significance analysis of microarrays (SAM) was performed to identify genes significantly differentially expressed across the four subtypes. These genes were trained by prediction analysis for microarrays (PAM) to achieve the lowest prediction error, which resulted in the 749-gene subtype-specific signature. This signature was applied to Tothill and Crijns cohorts, followed by consensus-based NMF analysis for molecular subtyping. Heatmaps were generated using GenePattern^58^.

### GSEA and ssGSEA

The Gene Set Enrichment Analysis (GSEA) software was downloaded from the Broad Institute GSEA portal and GSEA was performed as described^59^, using the VDGs as the input gene set. Single sample GSEA (ssGSEA) was applied to generate compound scores for VDGs, in which gene expression values were ranked for a given sample, and an enrichment score was calculated based on the normalized rank difference in Empirical Cumulative Distribution Functions (ECDF) of the genes in the signature and the remaining genes^60^.

### Survival analysis

The ssGSEA compound scores of VDGs were computed for each sample. The patients were dichotomized into a high-score and a low-score group, using the median VDGs score as the threshold value. Overall survival curves were calculated using the Kaplan–Meier method, and statistical significance was assessed using the log-rank test. The analyses were conducted with the R Bioconductor ‘survival’ package.

### Statistical analysis

Gene ontology and pathway analyses were performed in DAVID Bioinformatics Resources with GOTERM ALL categories^61^,^62^. Terms were defined as significantly enriched if they contained at least five counts and had a *P* value of < 0.001 and an FDR of < 5%. For microarray analyses, an empirical Bayes method was used to adjust *P* values for multiple comparisons. In all experiments, comparisons between two groups were based on two-sided Student’s t test, and one-way analysis of variance (ANOVA) was used to test for differences among more groups followed by post-hoc Tukey analysis for multiple comparisons. *P* values of < 0.05 were considered statistically significant.

## Additional Information

**How to cite this article**: Yin, X. *et al.* A VEGF-dependent gene signature enriched in mesenchymal ovarian cancer predicts patient prognosis. *Sci. Rep.* **6**, 31079; doi: 10.1038/srep31079 (2016).

## Supplementary Material

Supplementary Information
