# Supplementary material for: A VEGF-dependent gene signature enriched in mesenchymal ovarian cancer predicts patient prognosis
Source: Sci Rep. 2016 Aug 8;6:31079. doi: 10.1038/srep31079 (PMC4976329; doi:10.1038/srep31079)
Supplement: Supplementary Information [file srep31079-s1.pdf]

SUPPLEMENTARY INFORMATION

A VEGF-dependent gene signature enriched in mesenchymal ovarian cancer predicts patient prognosis

Xia Yin[1,2,3,#], Xiaojie Wang[4,#], Boqiang Shen[5,#], Ying Jing[1], Qing Li[2,3], Mei-Chun Cai[1,6], Zhuowei Gu[2,3], Qi Yang[7], Zhenfeng Zhang[1,6], Jin Liu[1,6], Hongxia Li[5], Wen Di[1,2,3,*], Guanglei Zhuang[1,3,*]

[1]State Key Laboratory of Oncogenes and Related Genes, Renji-Med X Clinical Stem Cell Research Center, Ren Ji Hospital, School of Medicine, Shanghai Jiao Tong University, Shanghai, China
[2]Department of Obstetrics and Gynecology, Ren Ji Hospital, School of Medicine, Shanghai Jiao Tong University, Shanghai, China
[3]Shanghai Key Laboratory of Gynecologic Oncology, Ren Ji Hospital, School of Medicine, Shanghai Jiao Tong University, Shanghai, China
[4]Department of Obstetrics and Gynecology, Shanghai General Hospital, Shanghai Jiao Tong University School of Medicine, Shanghai, China
[5]Department of Obstetrics and Gynecology, Beijing Shijitan Hospital, Capital Medical University, Beijing, China
[6]State Key Laboratory of Oncogenes and Related Genes, Shanghai Cancer Institute, Ren Ji Hospital, School of Medicine, Shanghai Jiao Tong University, Shanghai, China
[7]Lingyun Community Health Service Center of Xuhui District, Shanghai, China

[*]Corresponding author:
Guanglei Zhuang
Email: zhuangguanglei@gmail.com
or
Wen Di
Email: diwen163@163.com

[#]These authors contributed equally to this work.

Supplementary Figure 1.

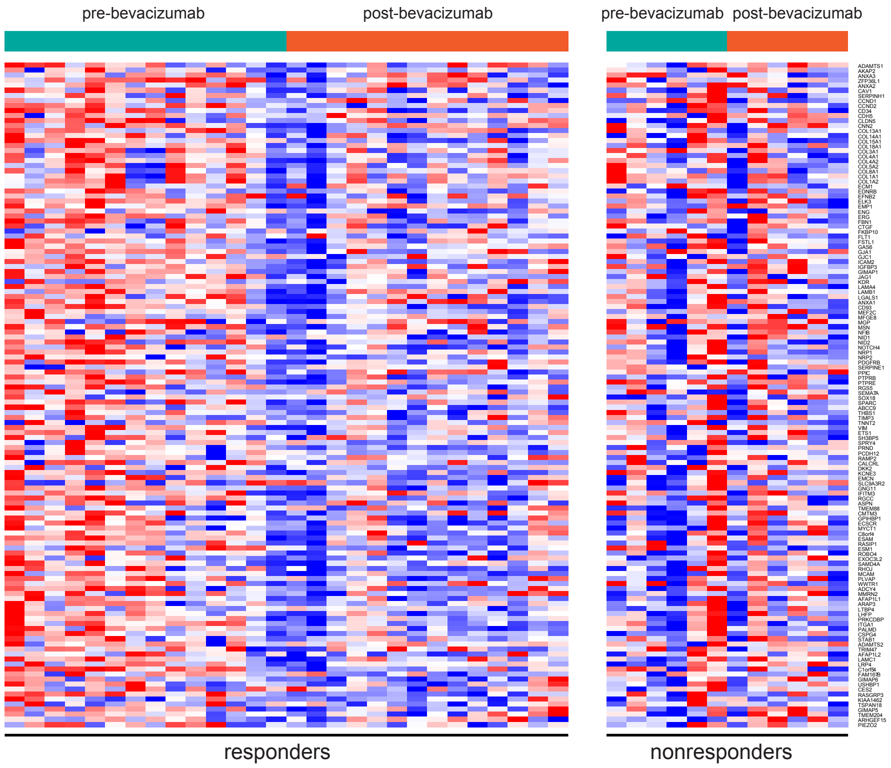

Supplementary Figure 1. Heatmap of the VDGs gene expression in serial clinical specimens collected from breast cancer patients treated with neoadjuvant bevacizumab.

Supplementary Figure 2.

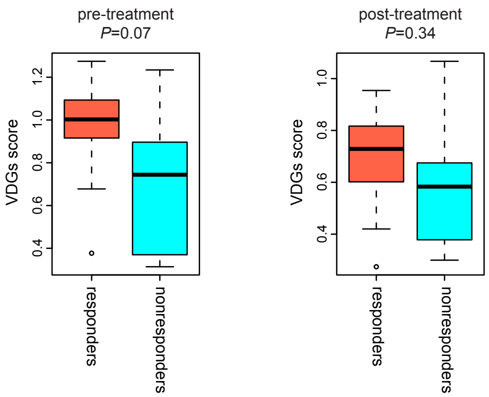

Supplementary Figure 2. Changes of the VDGs scores in serial clinical specimens collected from breast cancer patients treated with neoadjuvant bevacizumab.

Supplementary Figure 3.

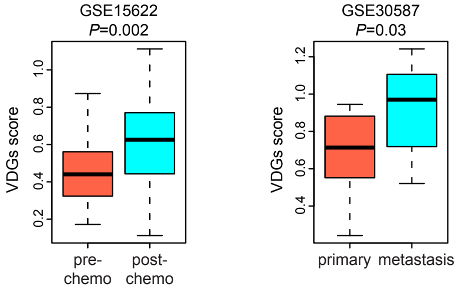

Supplementary Figure 3. Upregulation of the VDGs upon chemotherapy or tumor metastasis.

Supplementary Figure 4.

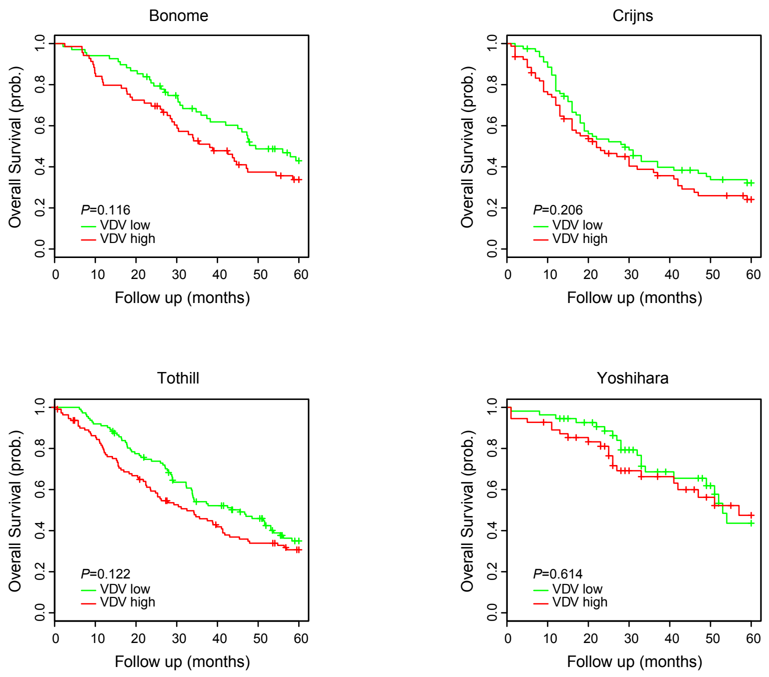

Supplementary Figure 4. Kaplan Meier curves for the two prognostic groups of HGS-OvCa expression profiles in Bonome, Crijns, Tothill and Yoshihara cohorts.

Supplementary Figure 5.

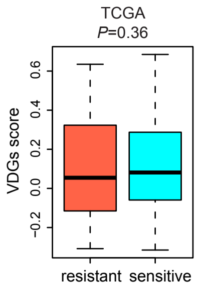

Supplementary Figure 5. VDGs scores in TCGA HGS-OvCa samples that are sensitive or resistant to chemotherapy.

Supplementary Table 1: Significantly downregulated genes upon anti-VEGF treatment identified by Affymatrix microarray

ProbeID	EntrezID	Symbol	logFC	adjusted P.Value
1438651_a_at	23796	Aplnr	-2.151909568	5.47E-06
1423516_a_at	18074	Nid2	-1.98770205	5.78E-05
1424051_at	12827	Col4a2	-1.768863442	6.12E-05
1447584_s_at	68632	Myct1	-1.376372768	6.12E-05
1419589_at	17064	Cd93	-1.840775932	0.000129365
1438855_x_at	21928	Tnfaip2	-1.103865293	0.000129365
1440244_at	13876	Erg	-1.201846845	0.000174433
1418939_at	15284	Hlx	-0.686066784	0.000174433
1449145_a_at	12389	Cav1	-0.980223963	0.000257162
1448755_at	12819	Col15a1	-1.554558889	0.000257162
1433956_at	12562	Cdh5	-1.801181041	0.000278724
1456768_a_at	105450	Mmrn2	-1.714248791	0.000278724
1451475_at	67784	Plxnd1	-1.090384158	0.000278724
1417818_at	97064	Wwtr1	-1.3867536	0.000298086
1428535_at	240185	9430020K01Rik	-1.07326449	0.000315812
1448213_at	16952	Anxa1	-1.227098907	0.000315812
1460330_at	11745	Anxa3	-1.034740008	0.000315812
1417420_at	12443	Ccnd1	-1.252502313	0.000315812
1448862_at	15896	Icam2	-1.182326911	0.000315812
1416808_at	18073	Nid1	-1.327986067	0.000315812
1423885_at	226519	Lamc1	-0.834945593	0.000336255
1416072_at	12490	Cd34	-1.736573598	0.000393246
1437451_at	68545	Ecscr	-1.082081611	0.000494587
1424807_at	16775	Lama4	-1.980297575	0.000538022
1442115_at	667742	Piezo2	-1.099481482	0.000590998
1441972_at	67786	6230424C14Rik	-1.087727213	0.000774671
1452163_at	23871	Ets1	-1.724773545	0.000840233
1438030_at	240168	Rasgrp3	-1.389732278	0.000840233
1450981_at	12798	Cnn2	-0.551061814	0.000910456
1420512_at	56811	Dkk2	-0.500383259	0.000910456
1425582_a_at	59308	Emcn	-1.972398819	0.000910456
1423771_at	109042	Prkcdbp	-0.73545026	0.000961593
1450958_at	17112	Tm4sf1	-1.178840956	0.000961593
1448797_at	13713	Elk3	-1.126745997	0.000970255
1415800_at	14609	Gja1	-2.08686601	0.000970255
1450414_at	18591	Pdgfb	-0.862642734	0.000970255
1449134_s_at	20728	Spic	-1.042466546	0.000970255

1449852_a_at	98878	Ehd4	-0.887929491	0.000975912
1427137_at	234673	Ces2e	-0.722062974	0.000984559
1426206_at	74144	Robo4	-1.450670144	0.000984559
1422866_at	12817	Col13a1	-0.850569453	0.000992079
1448943_at	18186	Nrp1	-1.249432048	0.000992079
1425681_a_at	26434	Prnd	-1.472048347	0.000992079
1448392_at	20692	Sparc	-1.650830826	0.000992079
1419186_a_at	20452	St8sia4	-0.63398788	0.000992079
1426348_at	12826	Col4a1	-1.455320245	0.001090748
1418448_at	20130	Rras	-0.586226767	0.001228711
1448942_at	66066	Gng11	-0.959486407	0.001264125
1419467_at	66864	Clec14a	-1.085464575	0.001319002
1418394_a_at	26364	Cd97	-0.574028438	0.001366839
1448729_a_at	18952	Sept4	-1.044491932	0.001476919
1451500_at	234395	Ushbp1	-0.978301253	0.001476919
1427891_at	231931	Gimap6	-0.926777698	0.001503134
1455251_at	109700	Itga1	-0.83496034	0.001503134
1436870_s_at	226250	Afap1l2	-0.917181868	0.001523409
1424375_s_at	107526	Gimap4	-1.377317564	0.001680323
1437928_at	53601	Pcdh12	-0.877911115	0.001680323
1456292_a_at	22352	Vim	-1.170099377	0.001680323
1421172_at	11489	Adam12	-0.52726011	0.001792079
1418237_s_at	12822	Col18a1	-0.888403898	0.001792079
1419833_s_at	106952	Arap3	-1.206509218	0.00181655
1436939_at	217012	Unc45b	-0.851242452	0.001882082
1428867_at	74463	Exoc3l2	-1.343032912	0.001955379
1425896_a_at	14118	Fbn1	-0.917950678	0.001955379
1448688_at	27205	Podxl	-1.331110736	0.001955379
1419638_at	13642	Efnb2	-0.737359964	0.002027324
1448471_a_at	13024	Ctla2a	-1.395705497	0.002073467
1421287_a_at	18613	Pecam1	-1.40883512	0.002076412
1449146_at	18132	Notch4	-1.180828961	0.002077909
1448594_at	22402	Wisp1	-0.923763109	0.00218884
1448864_at	20623	Snrk	-0.417774604	0.002195525
1416652_at	66695	Aspn	-0.939141407	0.002197597
1452250_a_at	12834	Col6a2	-0.813370209	0.002197597
1418090_at	84094	Plvap	-1.561824024	0.002231517
1424113_at	16777	Lamb1	-1.550663652	0.002342762
1438325_at	14013	Mecom	-0.531524706	0.002489425
1435436_at	13819	Epas1	-1.328162969	0.002534391
1460302_at	21825	Thbs1	-0.995885962	0.00260613
1417327_at	12390	Cav2	-0.696469683	0.002708616

1435701_at	224405	Cyyr1	-1.329521849	0.002725021
1460578_at	232237	Fgd5	-0.981311673	0.002954005
1428922_at	66873	Tril	-0.816197815	0.002954005
1420425_at	12142	Prdm1	-0.790588116	0.002997296
1448710_at	12767	Cxcr4	-0.728335517	0.003091639
1438658_a_at	13610	S1pr3	-1.075117394	0.003215576
1436233_at	237754	Btnl9	-1.35305526	0.003230744
1420361_at	18173	Slc11a1	-0.519161951	0.003230744
1449135_at	20672	Sox18	-1.206189032	0.003230744
1437347_at	13618	Ednrb	-1.265339477	0.003253814
1418547_at	21789	Tfpi2	-0.646456539	0.003378402
1452834_at	72446	Prr5l	-0.669240848	0.003450001
1442174_at	241556	Tspan18	-0.362507738	0.003450001
1422253_at	12813	Col10a1	-0.917169859	0.003561491
1453055_at	214968	Sema6d	-0.564192819	0.003561491
1451428_x_at	353156	Egfl7	-0.950823099	0.003691021
1418634_at	18128	Notch1	-0.519525153	0.003739529
1418703_at	56878	Rbms1	-0.435435778	0.003739529
1416238_at	21846	Tie1	-1.69755838	0.003739529
1460014_at	224840	Treml4	-0.62812487	0.003740579
1448316_at	68119	Cmtm3	-0.574926225	0.003769118
1420911_a_at	17304	Mfge8	-0.669166676	0.004072119
1444052_at	14257	Flt4	-0.71307982	0.004146813
1436698_x_at	407831	Tmem204	-1.518635792	0.004146813
1455164_at	12549	Arhgap31	-0.541497167	0.004217301
1419091_a_at	12306	Anxa2	-1.029384087	0.004245188
1415951_at	14230	Fkbp10	-0.515024502	0.004245188
1456288_at	327978	Slfn5	-0.508943573	0.004245188
1448590_at	12833	Col6a1	-0.864501349	0.004255744
1426288_at	228357	Lrp4	-0.428662826	0.004631235
1421375_a_at	20200	S100a6	-0.819297533	0.005001866
1435990_at	216725	Adamts2	-0.703063543	0.005082436
1424099_at	69590	Gpx8	-0.795998041	0.005082436
1423267_s_at	16402	Itga5	-0.49992845	0.005082436
1450644_at	12192	Zfp36l1	-0.679821769	0.005082436
1434248_at	18755	Prkch	-0.565103481	0.005088177
1454678_s_at	101351	Eogt	-0.499511739	0.005111458
1423341_at	121021	Cspg4	-0.572564014	0.005715603
1456061_at	243374	Gimap8	-0.734185796	0.005774215
1426784_at	217333	Trim47	-0.505255396	0.005837117
1436970_a_at	18596	Pdgfrb	-0.866821696	0.005853453
1425814_a_at	54598	Calcrl	-1.435892195	0.005911706

1455243_at	268936	Brpf3	-0.408544563	0.005941678
1418059_at	170757	Eltd1	-1.319391905	0.005941678
1419508_at	19766	Ripk1	-0.433046465	0.005941678
1419149_at	18787	Serpine1	-0.939839162	0.006167367
1451245_at	218763	Lrrc3b	-0.681533775	0.006177137
1421027_a_at	17260	Mef2c	-0.862748995	0.006377269
1423543_at	20947	Swap70	-0.544551917	0.006702128
1450843_a_at	12406	Serpinh1	-1.501444623	0.00716254
1435752_s_at	20928	Abcc9	-0.814647485	0.007202119
1454727_at	106877	Afap1l1	-0.657982842	0.007321355
1449495_at	19694	Reg3a	-1.788626454	0.007491872
1450716_at	11504	Adamts1	-0.905277206	0.007541693
1417839_at	12741	Cldn5	-0.47663715	0.007541693
1439441_x_at	50523	Lats2	-0.748999509	0.007626503
1415983_at	18826	Lcp1	-0.546860747	0.007626503
1422671_s_at	72560	Naalad2	-0.448359117	0.007626503
1422620_s_at	19012	Ppap2a	-0.931950724	0.00770933
1428016_a_at	69903	Rasip1	-0.869690454	0.008027903
1425840_a_at	20350	Sema3f	-0.990864958	0.00839891
1417595_at	17285	Meox1	-0.365812881	0.008415548
1418539_a_at	19267	Ptpre	-0.626089252	0.008431767
1436356_at	74480	Samd4	-0.398156164	0.008582114
1417271_a_at	13805	Eng	-0.932832879	0.008798296
1423571_at	13609	S1pr1	-0.441964316	0.009265706
1415855_at	17311	Kitl	-0.819195516	0.009267088
1435349_at	18187	Nrp2	-0.819202779	0.009267088
1453285_at	67020	Tmem88	-0.569401005	0.009745343
1440355_at	207474	Kctd12b	-0.748544967	0.009842686
1428808_at	243548	Prickle2	-0.515781408	0.009842686
1427168_a_at	12818	Col14a1	-0.351072224	0.00990829
1416630_at	15903	Id3	-0.971463092	0.009935897
1449379_at	16542	Kdr	-1.551923376	0.009935897
1448323_a_at	12111	Bgn	-0.845321248	0.009986505
1429035_at	71854	Dpep3	-0.393535515	0.009986505
1420942_s_at	19737	Rgs5	-1.842977564	0.009986505
1456532_at	71785	Pdgfd	-0.806593742	0.01030324
1439221_s_at	21939	Cd40	-0.668851566	0.010308254
1420804_s_at	17474	Clec4d	-0.38314729	0.010445257
1449988_at	16205	Gimap1	-0.375569342	0.010565431
1449094_at	14615	Gjc1	-0.709782463	0.010573991
1448259_at	14314	Fstl1	-1.087695715	0.010745081
1442075_at	102027	AI314604	-1.002441532	0.010761924

1418670_s_at	15530	Hspg2	-0.688818756	0.010761924
1428615_at	67168	Lpar6	-0.416864631	0.010761924
1422545_at	21385	Tbx2	-0.673432071	0.01107493
1439665_at	78134	Lpar4	-0.365194126	0.011095863
1435690_at	66356	Knop1	-0.363746435	0.011244405
1430976_a_at	78523	Mrpl9	-0.446282415	0.011244405
1449280_at	71690	Esm1	-2.698361852	0.011416426
1455965_at	240913	Adamts4	-0.470149604	0.011501991
1433796_at	71946	Endod1	-0.615842407	0.011501991
1425996_a_at	20585	Hltf	-0.499108737	0.01152792
1459903_at	20361	Sema7a	-0.36756883	0.011622205
1450883_a_at	12491	Cd36	-0.764840358	0.01166071
1418499_a_at	57442	Kcne3	-1.117314857	0.01166071
1416498_at	19038	Ppic	-1.061120842	0.01166071
1426670_at	11603	Agrn	-0.369066894	0.011877352
1416953_at	14219	Ctgf	-1.089086099	0.011877352
1436367_at	19263	Ptprb	-1.148383065	0.012016762
1429667_at	18996	Pou4f1	-0.499474538	0.012612033
1418726_a_at	21956	Tnnt2	-0.518302251	0.012612033
1448908_at	67916	Ppap2b	-1.31043891	0.01266554
1456385_x_at	212190	Ubxn10	-0.388481711	0.013465199
1417426_at	19073	Srgn	-0.697942063	0.013622197
1423498_at	79459	Aldoart2	-0.46431013	0.014083146
1419300_at	14254	Flt1	-1.249505693	0.014083146
1427327_at	231805	Pilra	-0.351267205	0.014328288
1448961_at	18828	Plscr2	-0.771831508	0.014543676
1427320_at	100044236	Copg2os2	-0.313936915	0.014588935
1436665_a_at	108075	Ltbp4	-0.959915387	0.014588935
1453022_at	68453	Gpihbp1	-0.543393226	0.014881271
1449860_at	75689	Higd1b	-0.528280364	0.014881271
1454633_at	75320	Etnk1	-0.467586977	0.015459504
1427679_at	16798	Lats1	-0.382949995	0.015518397
1427034_at	11421	Ace	-0.552160383	0.015638586
1420498_a_at	13132	Dab2	-0.506382905	0.015638586
1428420_a_at	74190	Exoc3l4	-0.517927203	0.015638586
1457745_at	319197	Gpr4	-0.403058977	0.015638586
1416268_at	23872	Ets2	-0.377000469	0.016424152
1457094_at	214547	She	-0.44259734	0.017014624
1416039_x_at	16007	Cyr61	-0.680251691	0.017078051
1418483_a_at	14594	Ggta1	-0.589794574	0.017078051
1455050_at	320736	Vstm4	-0.253852271	0.017638334
1435777_at	319622	Itpripl2	-0.553698336	0.017680986

1422811_at	26457	Slc27a1	-0.417777078	0.017708089
1427996_at	229600	BC028528	-0.704674319	0.01781239
1417185_at	110454	Ly6a	-0.896524047	0.017816347
1430522_a_at	53620	Vamp5	-0.506942307	0.017992235
1448510_at	13636	Efna1	-0.815377657	0.018453091
1433776_at	108927	Lhfp	-0.799744564	0.018970069
1418788_at	21687	Tek	-0.852661736	0.018970069
1425951_a_at	56620	Clec4n	-0.600933152	0.019113593
1456147_at	241230	St8sia6	-0.632401688	0.019113593
1428126_a_at	382423	Atxn7l3b	-0.486485877	0.019341455
1422445_at	16403	Itga6	-0.769967084	0.019341455
1417251_at	114301	Palmd	-0.295270073	0.019341455
1460039_at	243653	Clec1a	-0.466637517	0.020105979
1455627_at	12837	Col8a1	-0.849319378	0.020221071
1450355_a_at	12332	Capg	-0.575787023	0.02059759
1449168_a_at	11641	Akap2	-0.626436151	0.020633182
1420653_at	21803	Tgfb1	-0.70862931	0.020796626
1426246_at	19128	Pros1	-0.437335228	0.020925361
1416654_at	20530	Slc31a2	-0.346848768	0.022020454
1450839_at	27528	Nrep	-0.301870527	0.022122214
1448383_at	17387	Mmp14	-0.566032754	0.022186298
1444181_at	317757	Gimap5	-0.525844291	0.022226303
1441977_at	320378	9630023C09Rik	-0.476378581	0.022233104
1452421_at	15400	Hoxa3	-0.272593847	0.022361741
1437360_at	279653	Pcdh19	-0.609968996	0.022393797
1437576_at	72767	2810427A07Rik	-0.538222497	0.022428964
1440975_at	67622	Mxra7	-0.410756516	0.022507068
1429954_at	73149	Clec4a3	-0.407408706	0.022546605
1421826_at	54485	Dll4	-0.515491162	0.022690203
1460356_at	69524	Esam	-0.657147556	0.02307592
1452092_at	77590	Chst15	-0.810746636	0.023140639
1431099_at	15437	Hoxd8	-0.24795158	0.02364073
1448831_at	11601	Angpt2	-0.63306748	0.024459221
1456111_at	244853	Nxpe4	-0.916318227	0.024721018
1422631_at	11622	Ahr	-0.648133526	0.024868265
1418913_at	64918	Bhmt2	-0.636163502	0.024868265
1438511_a_at	66214	Rgcc	-1.420611011	0.024868265
1427318_s_at	226101	Myof	-0.371649902	0.025154436
1460197_a_at	117167	Steap4	-0.782792525	0.025154436
1438779_at	12828	Col4a3	-0.280726679	0.025188886
1423110_at	12843	Col1a2	-1.275361652	0.025223265
1417625_s_at	12778	Ackr3	-0.948811992	0.025595365

1435580_at	244895	C230081A13Rik	-0.328352758	0.025595365
1444816_at	258186	Olfr75-ps1	-0.358670537	0.025717124
1455587_at	230766	Fam167b	-0.926154101	0.025770686
1448416_at	17313	Mgp	-0.866742736	0.025770686
1418966_a_at	66686	Dcbld1	-0.469608994	0.025979139
1416357_a_at	84004	Mcam	-0.795325556	0.025979139
1437956_at	104709	Pik3r6	-0.429890337	0.025979139
1428954_at	65962	Slc9a3r2	-0.528566514	0.025979139
1447621_s_at	72512	Tmem173	-0.405174339	0.025979139
1434490_at	380713	Scarf1	-0.404154453	0.02610275
1428861_at	78749	Filip1l	-0.701515517	0.026210356
1427680_a_at	18028	Nfib	-0.927810892	0.026659748
1416123_at	12444	Ccnd2	-0.578128487	0.026799038
1427884_at	12825	Col3a1	-0.994887028	0.026818556
1422541_at	19274	Ptprm	-0.432420866	0.026969793
1447975_a_at	545261	Gm20748	-0.373118945	0.027202184
1429298_at	69219	Ddah1	-0.566968315	0.027236506
1448239_at	15368	Hmox1	-1.149979864	0.027236506
1418892_at	80837	Rhoj	-0.586034929	0.027247339
1456084_x_at	14264	Fmod	-0.703646864	0.027386643
1433741_at	12494	Cd38	-0.668119211	0.027632077
1424157_at	259300	Ehd2	-0.51294206	0.027632077
1458402_at	19286	Pts	-0.484536421	0.027842002
1429438_at	71458	Bcor	-0.356104854	0.027916792
1453448_at	76455	2310067E19Rik	-0.338361744	0.027953518
1435338_at	12571	Cdk6	-0.365213442	0.027953518
1440313_at	66848	Fuca2	-0.274226544	0.028079337
1437218_at	14268	Fn1	-0.489759175	0.028911605
1418102_at	15205	Hes1	-0.310442724	0.028911605
1415850_at	19414	Rasa3	-0.362636849	0.028911605
1417985_at	67122	Nrarp	-0.56248484	0.028940346
1444534_at	224648	Uhrf1bp1	-0.714141993	0.028943278
1452903_at	68176	Fam212a	-0.313555086	0.029016538
1423554_at	56316	Ggcx	-0.810017519	0.029016538
1434684_at	217835	Rin3	-0.395182707	0.029016538
1418157_at	13865	Nr2f1	-0.444087634	0.029064633
1455150_at	329152	Hecw2	-0.634034428	0.02913103
1451860_a_at	20128	Trim30a	-0.778267617	0.029170204
1422437_at	12832	Col5a2	-0.86381395	0.029201172
1454997_at	320183	Msrb3	-0.690113629	0.029610712
1437190_at	243659	Styk1	-0.408157727	0.029993287
1445669_at	24066	Spry4	-0.746872555	0.030397549

1448566_at	53945	Slc40a1	-0.921622362	0.030404641
1437271_at	56708	Clcf1	-0.334363847	0.030677078
1441618_at	214137	Arhgap29	-0.748272133	0.03120296
1435557_at	234686	Fhod1	-0.585341179	0.031318324
1459151_x_at	70110	Ifi35	-0.458850587	0.031899297
1453014_a_at	69162	Sec31a	-0.344365407	0.031899297
1419166_at	246787	Slc5a2	-0.354205828	0.031899297
1449335_at	21859	Timp3	-1.038470115	0.031899297
1448788_at	17470	Cd200	-0.407968852	0.032193535
1455494_at	12842	Col1a1	-1.088973456	0.032256315
1440144_x_at	78887	Sfi1	-0.582358415	0.032974843
1434070_at	16449	Jag1	-0.693637691	0.033346488
1424595_at	16456	F11r	-0.680363887	0.033734327
1434457_at	20684	Sp100	-0.43955706	0.033859686
1435825_at	11482	Acvrl1	-0.398711903	0.034196073
1440768_x_at	67340	1700052I22Rik	-0.352888151	0.035091453
1424768_at	109624	Cald1	-0.499637304	0.035446311
1429163_at	233651	Dchs1	-0.294999028	0.035509767
1450663_at	21826	Thbs2	-0.229491133	0.036245627
1449903_at	54698	Crtam	-0.337336978	0.036326436
1418379_s_at	78560	Gpr124	-0.439936681	0.036326436
1425959_x_at	27424	Klra16	-0.258637278	0.036326436
1451415_at	69068	1810011O10Rik	-1.002765948	0.036496356
1447882_x_at	71990	Ddx54	-0.867816792	0.036543151
1448983_at	66338	Cdrt4	-0.305479413	0.036683101
1452528_a_at	18089	Nkx2-3	-0.443434752	0.03672349
1420984_at	18559	Pctp	-0.509282075	0.037358825
1442902_at	217341	Qrich2	-0.40933324	0.037358825
1437661_at	239691	AU021092	-0.315013266	0.037768813
1424186_at	67896	Ccdc80	-0.579906209	0.037810386
1416323_at	66989	Kctd20	-0.351728892	0.038023522
1418098_at	104110	Adcy4	-0.41779352	0.038140184
1441228_at	381823	Apold1	-0.800700801	0.038356016
1435496_at	70564	Fam213a	-0.298181361	0.038570303
1420664_s_at	19124	Procr	-0.524227416	0.038585726
1434310_at	12168	Bmpr2	-0.353478859	0.038689416
1437936_at	434778	Ccdc160	-0.437541969	0.039257345
1434112_at	99633	Lphn2	-1.059375471	0.039689836
1421922_at	24056	Sh3bp5	-0.257701913	0.03971918
1460559_at	235041	Kank2	-0.378135972	0.040180151
1429203_at	73673	2410076I21Rik	-0.342701865	0.040319259
1419831_at	99996	AA416453	-0.350934072	0.040319259

1455439_a_at	16852	Lgals1	-0.839549078	0.04052019
1421356_at	21778	Tex9	-0.351174507	0.04052019
1434163_at	98582	Khdc1b	-0.305246495	0.040668267
1419137_at	58234	Shank3	-0.555613343	0.040668267
1456827_at	100416706	AA987161	-0.360828268	0.040670101
1442495_at	12211	Birc6	-1.058968202	0.040957326
1428759_s_at	67480	Cwc25	-0.594755885	0.041004019
1454086_a_at	16909	Lmo2	-0.3665779	0.041538248
1438081_at	328949	Mcc	-0.364208075	0.041538248
1416382_at	13032	Ctsc	-0.364798868	0.041561684
1422499_at	65970	Lima1	-0.428120712	0.041561684
1439834_at	100503924	Fcor	-0.649168727	0.041957186
1429177_x_at	20671	Sox17	-0.481378361	0.041957186
1417400_at	75646	Rai14	-0.7705404	0.042325504
1417399_at	14456	Gas6	-0.642004414	0.042618677
1420421_s_at	80782	Klrb1b	-0.258403605	0.04270609
1430848_a_at	103268	Cep57l1	-0.414909018	0.042724271
1440358_at	442801	Arhgef15	-0.447690375	0.042856963
1427009_at	16776	Lama5	-0.223969113	0.043051069
1434756_at	330593	5430421B17	-0.39136818	0.04321714
1439446_at	408058	BC048507	-0.484549424	0.04321714
1418187_at	54409	Ramp2	-0.722602153	0.04321714
1430453_a_at	12050	Bcl2l2	-0.398394409	0.043338938
1439540_at	224703	March2	-0.337353585	0.043566394
1417859_at	14457	Gas7	-0.336766764	0.043566394
1423754_at	66141	Ifitm3	-0.84174077	0.04429048
1450379_at	17698	Msn	-1.07644935	0.044403362
1435361_at	218877	Sema3g	-0.434515635	0.045523261
1448613_at	13601	Ecm1	-0.389514323	0.045582765
1425216_at	233079	Ffar2	-0.687484424	0.045582765
1434378_a_at	17122	Mxd4	-0.417373543	0.045582765
1424778_at	28193	Reep3	-0.333322221	0.045582765
1450199_a_at	192187	Stab1	-0.533024596	0.045582765
1452678_a_at	70266	Ccbl1	-0.357183643	0.046299584
1431781_at	106369	Ypel1	-0.372913108	0.046562125
1452063_at	73680	Zbtb8a	-0.402784611	0.046741484
1440922_at	77700	9130208D14Rik	-0.276158986	0.046809029
1452339_at	108153	Adamts7	-0.381760848	0.046809029
1452342_at	11787	Apbb2	-0.28630941	0.046809029
1416529_at	13730	Emp1	-0.863680893	0.046809029
1458268_s_at	16009	Igfbp3	-1.205116379	0.046809029
1439078_at	237010	Klhl4	-0.53736438	0.046809029

1434790_a_at	16993	Lta4h	-0.342021779	0.046957735
1418981_at	12364	Casp12	-0.486696593	0.046967166
1452474_a_at	109979	Art3	-0.497566064	0.047136923
1460640_at	11927	Atox1	-0.376893967	0.047136923
1444327_at	70790	Ubr5	-0.240153439	0.047136923
1429046_at	66313	Smurf2	-0.412161124	0.047311467
1431203_at	76816	Sdccag8	-0.408753266	0.047325957
1457034_at	218850	D14Abb1e	-0.540049695	0.047922794
1440075_at	59004	Pias4	-0.371747206	0.04803247
1416295_a_at	16186	Il2rg	-1.110691275	0.048372261
1431005_at	28109	D10Wsu102e	-0.263548561	0.04855328
1421217_a_at	16859	Lgals9	-0.968233949	0.049548003
1454604_s_at	269831	Tspan12	-0.658019416	0.04971969

Supplementary Table 2: Significantly downregulated genes upon anti-VEGF treatment identified by Agilent microarray

ProbeID	EntrezID	Symbol	logFC	adjusted P.Value
A_52_P257625	71690	Esm1	-3.5234187	1.01E-05
A_51_P204740	12490	Cd34	-2.554164927	1.01E-05
A_51_P503433	67916	Ppap2b	-2.494627097	1.01E-05
A_52_P365615	234673	Ces2e	-1.87111665	1.01E-05
A_51_P177371	26434	Prnd	-1.899327718	1.21E-05
A_51_P449824	74463	Exoc3l2	-1.294686108	0.000102845
A_51_P267754	15896	Icam2	-2.076194102	0.000104622
A_52_P253179	16009	Igfbp3	-1.960497066	0.000104622
A_51_P268563	13805	Eng	-1.723161318	0.000104622
A_51_P268439	84004	Mcam	-1.678268316	0.000104622
A_52_P209484	67020	Tmem88	-1.421179003	0.000104622
A_52_P515769	53601	Pcdh12	-1.419724687	0.000104622
A_52_P248604	12562	Cdh5	-2.007711795	0.000144832
A_51_P212420	16775	Lama4	-1.914245695	0.000144832
A_51_P332217	234395	Ushbp1	-1.446373432	0.000144832
A_51_P116906	223864	Rapgef3	-1.41681973	0.000208961
A_52_P375970	667742	Piezo2	-1.371567941	0.000210976
A_51_P502608	97064	Wwtr1	-1.130031382	0.000215481
A_52_P95544	380713	Scarf1	-0.899100443	0.00024807
A_51_P153124	59308	Emcn	-2.412692898	0.000254858
A_51_P138939	74144	Robo4	-1.644112188	0.000254858
A_51_P135802	17260	Mef2c	-0.888344078	0.000254858
A_51_P467224	14118	Fbn1	-0.720794451	0.000254858
A_51_P316553	16542	Kdr	-1.814137155	0.000263374
A_51_P463765	21859	Timp3	-1.448127213	0.000306943
A_51_P157042	14219	Ctgf	-1.91140925	0.000311572
A_52_P278064	121021	Cspg4	-1.4137881	0.000313389
A_51_P290576	20620	Plk2	-1.18402267	0.000321245
A_51_P414396	105450	Mmrn2	-2.348325504	0.000378113
A_51_P448325	69524	Esam	-0.934759156	0.000444244
A_52_P520859	14254	Flt1	-2.347848418	0.000478798
A_51_P244287	16777	Lamb1	-1.977527443	0.000478798
A_52_P13389	68632	Myct1	-1.118460099	0.000478798
A_52_P336958	240185	9430020K01Rik	-1.470809101	0.000550489
A_51_P238722	17064	Cd93	-2.349334685	0.000572077
A_51_P315666	18074	Nid2	-2.559507848	0.000586009
A_52_P499821	13876	Erg	-1.492760454	0.000587299

A_51_P507509	16205	Gimap1	-0.845802322	0.000660124
A_51_P157193	80837	Rhoj	-1.309769197	0.000672547
A_52_P195018	106952	Arap3	-1.732009474	0.00069684
A_52_P533809	15901	Id1	-2.19988604	0.000728456
A_51_P414653	84094	Plvap	-1.007802874	0.000885947
A_51_P383991	18952	Sept4	-0.922220405	0.000885947
A_52_P627357	13713	Elk3	-1.275531772	0.000925032
A_51_P215106	14314	Fstl1	-1.053629101	0.000925032
A_51_P480328	170757	Eltd1	-1.096359275	0.001016182
A_51_P139920	23945	Mgll	-1.333113986	0.00115964
A_51_P314517	80880	Kank3	-0.810150282	0.00115964
A_52_P635078	228357	Lrp4	-1.110280369	0.001273285
A_52_P459929	109700	Itga1	-0.690238556	0.001273285
A_51_P124254	12826	Col4a1	-1.900363839	0.001439813
A_51_P226269	66214	Rgcc	-2.145097504	0.001639461
A_51_P262766	12443	Ccnd1	-1.027550021	0.00193842
A_51_P336599	57442	Kcne3	-1.599182729	0.002087177
A_52_P93910	18187	Nrp2	-1.565322858	0.002114222
A_51_P366542	26364	Cd97	-0.998888172	0.002114222
A_51_P516860	217944	Rapgef5	-0.88997221	0.002114222
A_51_P197596	226250	Afap1l2	-0.889917067	0.002114222
A_52_P479249	21838	Thy1	-0.717412659	0.002114222
A_52_P1026777	68545	Ecscr	-2.08450497	0.002244579
A_51_P287069	12406	Serpinh1	-1.927551749	0.002273086
A_51_P316935	213393	8430408G22Rik	-0.88324553	0.002273086
A_51_P189104	19012	Ppap2a	-0.875062747	0.002273086
A_51_P502614	67603	Dusp6	-1.34329024	0.002948841
A_52_P573255	104445	Cdc42ep1	-1.467275535	0.002983874
A_51_P268843	69903	Rasip1	-1.11276258	0.003117417
A_51_P159792	12819	Col15a1	-1.274679843	0.003286124
A_51_P180974	109042	Prkcdbp	-1.260082831	0.003374226
A_52_P410765	20361	Sema7a	-0.786342664	0.003374226
A_52_P363951	21825	Thbs1	-0.656006742	0.004121623
A_51_P181705	19270	Ptprg	-0.887939158	0.004518007
A_51_P407227	231931	Gimap6	-0.983591736	0.004818614
A_52_P3723	18028	Nfib	-0.768449042	0.004818614
A_51_P183571	18787	Serpine1	-0.861774611	0.005206923
A_51_P427516	56229	Thsd1	-0.846915999	0.005206923
A_51_P149699	14789	Leprel2	-0.66077907	0.005206923
A_51_P126198	13143	Dapk2	-0.908506889	0.00566962
A_52_P571290	69202	Ptms	-0.957643203	0.005830962
A_51_P405397	13601	Ecm1	-0.926797062	0.005830962

A_52_P282058	12837	Col8a1	-1.341049402	0.005856304
A_52_P305307	24056	Sh3bp5	-0.799230916	0.005856304
A_51_P427663	12798	Cnn2	-0.637965942	0.005856304
A_51_P176365	317757	Gimap5	-0.607811235	0.005856304
A_51_P431087	20692	Sparc	-2.038881096	0.006482914
A_51_P136355	66066	Gng11	-1.592545137	0.006482914
A_51_P220162	18131	Notch3	-0.845408185	0.006709707
A_52_P434549	56215	Acin1	-1.076830889	0.006722012
A_52_P340073	13642	Efnb2	-1.012539635	0.00689621
A_51_P191700	12822	Col18a1	-1.622745113	0.007331834
A_51_P434670	19317	Qk	-0.575038805	0.007331834
A_52_P194250	23871	Ets1	-1.044138556	0.007365139
A_51_P216965	14230	Fkbp10	-0.737914525	0.007434343
A_51_P295192	18035	Nfkbia	-0.74516925	0.007548727
A_52_P343627	63954	Rbp7	-2.356635222	0.007813548
A_51_P282508	11853	Rhoc	-0.733813986	0.008730533
A_51_P303739	106877	Afap1l1	-1.276349931	0.009052552
A_51_P325904	16324	Inhbb	-1.029390697	0.009133721
A_52_P198435	240168	Rasgrp3	-1.100835524	0.009159181
A_52_P62037	12306	Anxa2	-0.826736163	0.009245973
A_52_P241676	65962	Slc9a3r2	-0.557198157	0.009245973
A_51_P188271	70445	Cd248	-0.854054379	0.009256931
A_52_P274496	241556	Tspan18	-1.007233585	0.009338009
A_51_P491350	12827	Col4a2	-1.529298782	0.009434106
A_51_P321886	68119	Cmtm3	-0.802048919	0.00953324
A_51_P472292	29817	Igfbp7	-1.30135906	0.009909534
A_52_P326657	230766	Fam167b	-0.857119412	0.009909534
A_51_P338262	21956	Tnnt2	-0.512490166	0.010020514
A_51_P144264	16598	Klf2	-1.590995666	0.010209281
A_51_P161086	19038	Ppic	-1.606214092	0.010553945
A_51_P392687	22352	Vim	-1.248367001	0.010553945
A_51_P255682	17304	Mfge8	-1.123563332	0.010553945
A_51_P229911	104110	Adcy4	-0.884248901	0.010553945
A_51_P450573	21813	Tgfbr2	-0.703449381	0.010553945
A_51_P215475	19263	Ptprb	-1.794722579	0.010573122
A_52_P306305	11641	Akap2	-1.037995686	0.010573122
A_52_P309381	12628	Cfh	-1.235874522	0.010911881
A_51_P182303	12843	Col1a2	-1.050834799	0.011010035
A_52_P204311	24066	Spry4	-0.859821257	0.011010035
A_51_P361220	14366	Fzd4	-0.508179836	0.011614121
A_51_P300378	52357	Wwc2	-0.594054929	0.012570453
A_51_P283590	16952	Anxa1	-1.208524048	0.012764578

A_51_P128876	66141	Ifitm3	-0.73457	0.012764578
A_51_P181772	19267	Ptpre	-0.697918065	0.012764578
A_51_P489192	50706	Postn	-1.086554918	0.012836628
A_52_P263095	66395	Ahnak	-0.977217399	0.012932062
A_51_P171883	114301	Palmd	-0.723939588	0.013516677
A_52_P111031	219228	Pcdh17	-2.043518085	0.01398294
A_51_P420276	67448	Plxdc2	-0.894639165	0.014306257
A_51_P189814	12741	Cldn5	-0.581431743	0.015688241
A_52_P558411	17698	Msn	-0.605284149	0.016262296
A_52_P525107	12842	Col1a1	-1.359360469	0.016325132
A_52_P581138	329506	Ctdspl2	-0.684241136	0.016325132
A_51_P403536	108075	Ltbp4	-0.606189744	0.016325132
A_52_P634090	16449	Jag1	-0.993032804	0.016618403
A_51_P426270	17313	Mgp	-1.409899379	0.016645463
A_51_P232371	192187	Stab1	-1.060801609	0.016645463
A_51_P235801	12192	Zfp36l1	-0.886253925	0.016645463
A_52_P53906	12444	Ccnd2	-0.633744452	0.016906716
A_51_P227222	216725	Adamts2	-1.05577319	0.016934231
A_51_P317141	12824	Col2a1	-1.06937083	0.016967976
A_51_P241068	56811	Dkk2	-0.903186299	0.017299938
A_52_P107571	66880	Rsrc1	-0.761655269	0.017299938
A_51_P248044	16779	Lamb2	-0.713678262	0.017482059
A_51_P277536	54409	Ramp2	-0.949169767	0.017653228
A_51_P469285	18186	Nrp1	-0.983989348	0.01818965
A_51_P209327	30878	Apln	-0.408632115	0.018297235
A_51_P451032	11745	Anxa3	-0.963264418	0.018708453
A_52_P443846	67533	Ppfibp1	-0.650857252	0.018708453
A_51_P415475	114255	Dok4	-0.542325792	0.018708453
A_52_P643165	74480	Samd4	-0.484335679	0.019136406
A_51_P326529	12818	Col14a1	-0.962294375	0.019611118
A_52_P288251	407831	Tmem204	-0.679999646	0.02079697
A_51_P291417	21824	Thbd	-1.088998023	0.022405345
A_52_P136782	19737	Rgs5	-0.709114023	0.023835244
A_51_P388847	106766	Stap2	-0.400756977	0.024559929
A_51_P472274	20672	Sox18	-1.398099761	0.024598337
A_51_P247883	12832	Col5a2	-0.505542283	0.024598337
A_51_P225186	54598	Calcrl	-1.530750765	0.026414028
A_51_P502580	94092	Trim16	-0.606728858	0.026414028
A_51_P195875	18132	Notch4	-1.145544466	0.02706915
A_52_P285041	14615	Gjc1	-0.493626191	0.02706915
A_51_P339943	18073	Nid1	-2.069938004	0.027331208
A_51_P174407	229600	BC028528	-2.064948234	0.027566051

A_52_P577662	13618	Ednrb	-0.621788106	0.028112203
A_51_P197528	100041546	Ly6c2	-1.236999251	0.029211217
A_52_P174915	14609	Gja1	-0.95242014	0.029950613
A_51_P332075	218952	Fermt2	-0.712683748	0.030165238
A_51_P373163	330662	Dock1	-0.492020106	0.030165238
A_51_P172054	14456	Gas6	-0.891917762	0.031475116
A_51_P366344	21804	Tgfb1i1	-0.435554646	0.031609665
A_51_P336833	11770	Fabp4	-1.530144425	0.031685311
A_51_P173043	13654	Egr2	-0.548316609	0.031685311
A_51_P112308	69068	1810011O10Rik	-1.859632808	0.032448819
A_51_P268234	76527	Il34	-0.434633513	0.033071407
A_51_P450527	21345	Tagln	-0.794805886	0.034234432
A_51_P343739	228836	Dlgap4	-0.483671186	0.034234432
A_51_P139651	18127	Nos3	-0.50558215	0.034582935
A_52_P69506	442801	Arhgef15	-0.766172208	0.035221187
A_52_P94874	14683	Gnas	-0.759748541	0.036845246
A_51_P213544	16600	Klf4	-0.707488692	0.03808476
A_52_P1165070	24059	Slco2a1	-0.625613562	0.03808476
A_51_P123745	216892	Spns2	-0.510517357	0.03808476
A_51_P142813	66695	Aspn	-0.952240243	0.038739659
A_51_P476018	20680	Sox7	-0.827929617	0.038739659
A_52_P489295	11504	Adamts1	-0.507513395	0.038739659
A_51_P331328	68453	Gpihbp1	-0.46628776	0.038739659
A_51_P361678	68178	Cgnl1	-0.887313015	0.039473076
A_52_P306845	12389	Cav1	-0.760241194	0.0398062
A_51_P358484	270190	Ephb1	-0.688438918	0.040063338
A_52_P10793	19242	Ptn	-0.605513511	0.040063338
A_51_P150964	18596	Pdgfrb	-1.277755558	0.040237899
A_51_P328850	20928	Abcc9	-0.57301395	0.040237899
A_51_P115441	22240	Dpysl3	-0.553999728	0.040237899
A_51_P199354	226519	Lamc1	-0.882638376	0.04171089
A_52_P120037	13730	Emp1	-0.840815169	0.043131888
A_51_P505868	108927	Lhfp	-0.414340549	0.043830136
A_51_P489522	13025	Ctla2b	-1.680544148	0.044325234
A_51_P161890	14132	Fcgrt	-0.653627495	0.04668972
A_51_P515605	12825	Col3a1	-1.411978831	0.047626041
A_51_P465281	16852	Lgals1	-0.643672672	0.048079964
A_51_P240374	12817	Col13a1	-1.415720076	0.048635501
A_51_P282523	78560	Gpr124	-0.683690325	0.048635501
A_52_P471395	15980	Ifngr2	-0.573582204	0.048635501
A_51_P466270	15203	Heph	-0.440690266	0.048635501
A_51_P180747	13024	Ctla2a	-2.323077705	0.04936893

A_51_P147684	11819	Nr2f2	-0.620979418	0.04936893
A_51_P437176	217333	Trim47	-0.508796652	0.04936893

Supplementary Table 3: Genes in the VDGs

GeneSymbol Mouse	GeneSymbol Human
Abcc9	ABCC9
Adamts1	ADAMTS1
Adamts2	ADAMTS2
Adcy4	ADCY4
Gpr124	ADGRA2
Cd97	ADGRE5
Eltd1	ADGRL4
Afap1l1	AFAP1L1
Afap1l2	AFAP1L2
Akap2	AKAP2
Anxa1	ANXA1
Anxa2	ANXA2
Anxa3	ANXA3
Arap3	ARAP3
Arhgef15	ARHGEF15
Aspn	ASPN
BC028528	C1orf54
1810011O10Rik	C8orf4
Calcrl	CALCRL
Cav1	CAV1
Ccnd1	CCND1
Ccnd2	CCND2
Cd34	CD34
Cd93	CD93
Cdh5	CDH5
Ces2e	CES2
Cldn5	CLDN5
Cmtm3	CMTM3
Cnn2	CNN2
Col13a1	COL13A1
Col14a1	COL14A1
Col15a1	COL15A1
Col18a1	COL18A1
Col1a1	COL1A1
Col1a2	COL1A2
Col3a1	COL3A1
Col4a1	COL4A1
Col4a2	COL4A2

Col5a2	COL5A2
Col8a1	COL8A1
Cspg4	CSPG4
Ctgf	CTGF
Dkk2	DKK2
Ecm1	ECM1
Ecscr	ECSCR
Ednrb	EDNRB
Efnb2	EFNB2
Elk3	ELK3
Emcn	EMCN
Emp1	EMP1
Eng	ENG
Erg	ERG
Esam	ESAM
Esm1	ESM1
Ets1	ETS1
Exoc3l2	EXOC3L2
Fam167b	FAM167B
Fbn1	FBN1
Fkbp10	FKBP10
Flt1	FLT1
Fstl1	FSTL1
Gas6	GAS6
Gimap1	GIMAP1
Gimap5	GIMAP5
Gimap6	GIMAP6
Gja1	GJA1
Gjc1	GJC1
Gng11	GNG11
Gpihbp1	GPIHBP1
Icam2	ICAM2
Ifitm3	IFITM3
Igfbp3	IGFBP3
Itga1	ITGA1
Jag1	JAG1
Kcne3	KCNE3
Kdr	KDR
9430020K01Rik	KIAA1462
Lama4	LAMA4
Lamb1	LAMB1
Lamc1	LAMC1

Lgals1	LGALS1
Lhfp	LHFP
Lrp4	LRP4
Ltbp4	LTBP4
Mcam	MCAM
Mef2c	MEF2C
Mfge8	MFGE8
Mgp	MGP
Mmrn2	MMRN2
Msn	MSN
Myct1	MYCT1
Nfib	NFIB
Nid1	NID1
Nid2	NID2
Notch4	NOTCH4
Nrp1	NRP1
Nrp2	NRP2
Palmd	PALMD
Pcdh12	PCDH12
Pdgfrb	PDGFRB
Piezo2	PIEZO2
Ppap2a	PLPP1
Ppap2b	PLPP3
Plvap	PLVAP
Ppic	PPIC
Prkcdbp	PRKCDBP
Prnd	PRND
Ptprb	PTPRB
Ptpre	PTPRE
Ramp2	RAMP2
Rasgrp3	RASGRP3
Rasip1	RASIP1
Rgcc	RGCC
Rgs5	RGS5
Rhoj	RHOJ
Robo4	ROBO4
Samd4	SAMD4A
Scarf1	SCARF1
Sema7a	SEMA7A
Spet4	SEPT4
Serpine1	SERPINE1
Serpinh1	SERPINH1

Sh3bp5	SH3BP5
Slc9a3r2	SLC9A3R2
Sox18	SOX18
Sparc	SPARC
Spry4	SPRY4
Stab1	STAB1
Thbs1	THBS1
Timp3	TIMP3
Tmem204	TMEM204
Tmem88	TMEM88
Tnnt2	TNNT2
Trim47	TRIM47
Tspan18	TSPAN18
Ushbp1	USHBP1
Vim	VIM
Wwtr1	WWTR1
Zfp36l1	ZFP36L1

Supplementary Table 4: Gene Ontology terms overpresented in VDGs

Term	P-Value	FDR
GO:0001944~vasculature development	6.93E-22	1.13E-18
GO:0001568~blood vessel development	6.79E-21	1.10E-17
GO:0048513~organ development	2.25E-20	3.65E-17
GO:0048731~system development	5.80E-19	9.41E-16
GO:0048856~anatomical structure development	1.11E-18	1.81E-15
GO:0007275~multicellular organismal development	2.58E-17	4.18E-14
GO:0032502~developmental process	1.45E-16	1.78E-13
GO:0048514~blood vessel morphogenesis	3.29E-16	5.44E-13
GO:0009653~anatomical structure morphogenesis	1.43E-15	2.34E-12
GO:0001525~angiogenesis	2.71E-15	4.33E-12
GO:0007155~cell adhesion	2.20E-14	3.57E-11
GO:0022610~biological adhesion	2.29E-14	3.71E-11
GO:0048646~anatomical structure formation involved in morpho	5.75E-12	9.34E-09
GO:0030154~cell differentiation	3.94E-11	6.40E-08
GO:0030198~extracellular matrix organization	3.96E-11	6.43E-08
GO:0048869~cellular developmental process	1.41E-10	2.29E-07
GO:0032501~multicellular organismal process	8.44E-10	1.37E-06
GO:0030199~collagen fibril organization	2.51E-09	4.07E-06
GO:0042127~regulation of cell proliferation	2.72E-09	4.41E-06
GO:0043062~extracellular structure organization	7.48E-09	1.21E-05
GO:0009888~tissue development	1.39E-08	2.25E-05
GO:0050793~regulation of developmental process	1.77E-08	2.87E-05
GO:0051270~regulation of cell motion	4.96E-08	8.05E-05
GO:0030334~regulation of cell migration	1.08E-06	0.001754246
GO:0048870~cell motility	1.14E-06	0.00185751
GO:0051674~localization of cell	1.14E-06	0.00185751
GO:0032879~regulation of localization	1.78E-06	0.002896651
GO:0040011~locomotion	1.92E-06	0.003124147
GO:0016337~cell-cell adhesion	2.31E-06	0.003746062
GO:0016477~cell migration	2.31E-06	0.003746062
GO:0009887~organ morphogenesis	2.68E-06	0.004359111
GO:0040012~regulation of locomotion	3.43E-06	0.005563935
GO:0006928~cell motion	6.30E-06	0.010228714
GO:0045446~endothelial cell differentiation	6.81E-06	0.011062852
GO:0001501~skeletal system development	1.01E-05	0.016471162
GO:0031589~cell-substrate adhesion	1.41E-05	0.022819403
GO:0048522~positive regulation of cellular process	1.62E-05	0.026271578
GO:0051239~regulation of multicellular organismal process	1.70E-05	0.02758299

GO:0001570~vasculogenesis	1.87E-05	0.030345668
GO:0001763~morphogenesis of a branching structure	2.88E-05	0.046757859
GO:0048519~negative regulation of biological process	2.95E-05	0.047934727
GO:0044420~extracellular matrix part	2.63E-23	3.28E-20
GO:0031012~extracellular matrix	2.55E-22	3.18E-19
GO:0005578~proteinaceous extracellular matrix	4.95E-22	6.20E-19
GO:0044421~extracellular region part	8.47E-18	1.06E-14
GO:0005604~basement membrane	2.19E-17	2.73E-14
GO:0005581~collagen	7.56E-14	9.44E-11
GO:0005576~extracellular region	6.61E-12	8.27E-09
GO:0005886~plasma membrane	7.94E-10	9.92E-07
GO:0044459~plasma membrane part	2.25E-08	2.82E-05
GO:0005201~extracellular matrix structural constituent	2.73E-15	3.66E-12
GO:0019838~growth factor binding	2.21E-11	2.92E-08
GO:0005198~structural molecule activity	3.29E-10	4.34E-07
GO:0005515~protein binding	3.93E-09	5.19E-06
GO:0005021~vascular endothelial growth factor receptor activity	2.16E-07	2.85E-04
GO:0048407~platelet-derived growth factor binding	1.00E-06	0.00132035
GO:0005178~integrin binding	5.46E-06	0.007213111
GO:0005518~collagen binding	7.23E-06	0.009549173
GO:0005509~calcium ion binding	1.78E-05	0.023488455

Supplementary Table 5: BRCA mutation status of TCGA samples

ID	BRCA1	BRCA2
TCGA-04-1331-01	NaN	C711*
TCGA-04-1332-01	NaN	NaN
TCGA-04-1336-01	NaN	T1738Ifs*2
TCGA-04-1337-01	NaN	NaN
TCGA-04-1338-01	NaN	NaN
TCGA-04-1342-01	NaN	NaN
TCGA-04-1343-01	NaN	NaN
TCGA-04-1346-01	NaN	NaN
TCGA-04-1347-01	NaN	NaN
TCGA-04-1348-01	NaN	NaN
TCGA-04-1349-01	NaN	NaN
TCGA-04-1350-01	NaN	NaN
TCGA-04-1356-01	N723Ifs*13	NaN
TCGA-04-1357-01	Q1538*	NaN
TCGA-04-1361-01	NaN	NaN
TCGA-04-1362-01	NaN	NaN
TCGA-04-1364-01	NaN	NaN
TCGA-04-1365-01	NaN	NaN
TCGA-04-1367-01	NaN	E294*
TCGA-04-1514-01	NaN	NaN
TCGA-04-1517-01	NaN	NaN
TCGA-04-1525-01	NaN	NaN
TCGA-04-1530-01	NaN	NaN
TCGA-04-1542-01	NaN	NaN
TCGA-09-0366-01	NaN	NaN
TCGA-09-0369-01	NaN	NaN
TCGA-09-1659-01	NaN	NaN
TCGA-09-1661-01	NaN	NaN
TCGA-09-1662-01	NaN	NaN
TCGA-09-1665-01	NaN	NaN
TCGA-09-1666-01	NaN	NaN
TCGA-09-1669-01	E1346Kfs*20	NaN
TCGA-09-2044-01	NaN	NaN
TCGA-09-2045-01	Q1779Nfs*14	NaN
TCGA-09-2049-01	NaN	NaN
TCGA-09-2050-01	NaN	S1882*
TCGA-09-2051-01	Q1756Pfs*74	NaN
TCGA-09-2053-01	NaN	NaN

TCGA-09-2056-01	NaN	NaN
TCGA-10-0926-01	NaN	NaN
TCGA-10-0927-01	NaN	NaN
TCGA-10-0928-01	NaN	NaN
TCGA-10-0930-01	NaN	NaN
TCGA-10-0931-01	E23Vfs*17	NaN
TCGA-10-0933-01	NaN	NaN
TCGA-10-0934-01	NaN	NaN
TCGA-10-0935-01	NaN	NaN
TCGA-10-0937-01	NaN	NaN
TCGA-10-0938-01	NaN	NaN
TCGA-13-0714-01	NaN	NaN
TCGA-13-0717-01	NaN	NaN
TCGA-13-0720-01	NaN	NaN
TCGA-13-0723-01	NaN	NaN
TCGA-13-0724-01	NaN	NaN
TCGA-13-0726-01	NaN	R2394*
TCGA-13-0727-01	NaN	NaN
TCGA-13-0730-01	R1835*	NaN
TCGA-13-0751-01	NaN	NaN
TCGA-13-0755-01	NaN	NaN
TCGA-13-0760-01	NaN	NaN
TCGA-13-0761-01	X1495_splice	NaN
TCGA-13-0762-01	NaN	NaN
TCGA-13-0765-01	NaN	NaN
TCGA-13-0791-01	NaN	NaN
TCGA-13-0792-01	NaN	E1143D
TCGA-13-0793-01	NaN	G3086Tfs*24
TCGA-13-0795-01	NaN	NaN
TCGA-13-0800-01	NaN	NaN
TCGA-13-0804-01	C47W	NaN
TCGA-13-0807-01	NaN	NaN
TCGA-13-0883-01	Q1756Pfs*74	NaN
TCGA-13-0884-01	NaN	NaN
TCGA-13-0885-01	NaN	K1406Nfs*3
TCGA-13-0886-01	NaN	S1982Rfs*22
TCGA-13-0887-01	NaN	NaN
TCGA-13-0889-01	NaN	NaN
TCGA-13-0890-01	NaN	S1230Lfs*9
TCGA-13-0891-01	NaN	NaN
TCGA-13-0893-01	R504*	NaN
TCGA-13-0894-01	NaN	NaN

TCGA-13-0897-01	NaN	NaN
TCGA-13-0899-01	NaN	NaN
TCGA-13-0900-01	NaN	N257Kfs*17
TCGA-13-0903-01	R504Vfs*28	NaN
TCGA-13-0904-01	NaN	NaN
TCGA-13-0905-01	NaN	NaN
TCGA-13-0906-01	NaN	NaN
TCGA-13-0910-01	NaN	NaN
TCGA-13-0911-01	NaN	NaN
TCGA-13-0912-01	NaN	NaN
TCGA-13-0913-01	NaN	E1857Nfs*2
TCGA-13-0916-01	NaN	NaN
TCGA-13-0919-01	NaN	NaN
TCGA-13-0920-01	NaN	NaN
TCGA-13-0923-01	NaN	NaN
TCGA-13-0924-01	NaN	NaN
TCGA-13-1403-01	NaN	NaN
TCGA-13-1404-01	NaN	NaN
TCGA-13-1405-01	NaN	NaN
TCGA-13-1407-01	NaN	NaN
TCGA-13-1408-01	E23Vfs*17	NaN
TCGA-13-1409-01	NaN	NaN
TCGA-13-1410-01	NaN	NaN
TCGA-13-1411-01	NaN	NaN
TCGA-13-1412-01	NaN	NaN
TCGA-13-1477-01	NaN	NaN
TCGA-13-1481-01	NaN	S2697Kfs*31
TCGA-13-1482-01	NaN	NaN
TCGA-13-1483-01	NaN	NaN
TCGA-13-1484-01	NaN	NaN
TCGA-13-1487-01	NaN	NaN
TCGA-13-1488-01	NaN	NaN
TCGA-13-1489-01	N1265Kfs*4	NaN
TCGA-13-1491-01	NaN	NaN
TCGA-13-1492-01	NaN	NaN
TCGA-13-1494-01	X45_splice	NaN
TCGA-13-1495-01	NaN	NaN
TCGA-13-1496-01	NaN	NaN
TCGA-13-1497-01	NaN	NaN
TCGA-13-1498-01	NaN	S1982Rfs*22
TCGA-13-1499-01	NaN	S1982Rfs*22
TCGA-13-1501-01	NaN	NaN

TCGA-13-1504-01	NaN	NaN
TCGA-13-1505-01	NaN	NaN
TCGA-13-1506-01	NaN	NaN
TCGA-13-1507-01	NaN	NaN
TCGA-13-1509-01	NaN	NaN
TCGA-13-1510-01	NaN	NaN
TCGA-13-1512-01	D825Efs*21	K3326*
TCGA-13-2060-01	NaN	NaN
TCGA-20-0987-01	NaN	NaN
TCGA-20-0990-01	NaN	NaN
TCGA-20-0991-01	NaN	NaN
TCGA-23-1021-01	NaN	NaN
TCGA-23-1022-01	NaN	NaN
TCGA-23-1023-01	NaN	NaN
TCGA-23-1024-01	NaN	NaN
TCGA-23-1026-01	G813Dfs*2	K3326*
TCGA-23-1027-01	E23Vfs*17	NaN
TCGA-23-1028-01	NaN	NaN
TCGA-23-1030-01	NaN	T1354M
TCGA-23-1031-01	NaN	NaN
TCGA-23-1032-01	NaN	NaN
TCGA-23-1110-01	NaN	NaN
TCGA-23-1116-01	NaN	NaN
TCGA-23-1117-01	NaN	NaN
TCGA-23-1118-01	E23Vfs*17	NaN
TCGA-23-1120-01	NaN	P3278Lfs*35
TCGA-23-1122-01	Q1756Pfs*74	NaN
TCGA-23-1123-01	NaN	NaN
TCGA-23-1124-01	NaN	NaN
TCGA-23-2072-01	NaN	NaN
TCGA-23-2077-01	Q1756Pfs*74	NaN
TCGA-23-2078-01	E23Vfs*17	NaN
TCGA-23-2079-01	E23Vfs*17	NaN
TCGA-23-2081-01	Q1756Pfs*74	NaN
TCGA-24-0966-01	NaN	NaN
TCGA-24-0968-01	NaN	NaN
TCGA-24-0970-01	NaN	NaN
TCGA-24-0975-01	NaN	X211_splice
TCGA-24-0979-01	NaN	NaN
TCGA-24-0980-01	NaN	NaN
TCGA-24-0982-01	NaN	NaN
TCGA-24-1103-01	NaN	K1638E

TCGA-24-1104-01	NaN	NaN
TCGA-24-1105-01	NaN	NaN
TCGA-24-1413-01	NaN	NaN
TCGA-24-1416-01	NaN	NaN
TCGA-24-1417-01	NaN	N1706Lfs*5
TCGA-24-1418-01	NaN	NaN
TCGA-24-1419-01	NaN	NaN
TCGA-24-1422-01	NaN	NaN
TCGA-24-1423-01	NaN	NaN
TCGA-24-1424-01	NaN	NaN
TCGA-24-1425-01	NaN	NaN
TCGA-24-1426-01	NaN	NaN
TCGA-24-1427-01	NaN	NaN
TCGA-24-1428-01	NaN	NaN
TCGA-24-1431-01	NaN	NaN
TCGA-24-1434-01	NaN	NaN
TCGA-24-1435-01	NaN	NaN
TCGA-24-1436-01	NaN	NaN
TCGA-24-1463-01	NaN	I605Nfs*11
TCGA-24-1464-01	NaN	NaN
TCGA-24-1466-01	NaN	NaN
TCGA-24-1469-01	NaN	NaN
TCGA-24-1470-01	T1677Ifs*2	NaN
TCGA-24-1471-01	NaN	NaN
TCGA-24-1474-01	NaN	NaN
TCGA-24-1544-01	NaN	NaN
TCGA-24-1545-01	NaN	NaN
TCGA-24-1548-01	NaN	NaN
TCGA-24-1549-01	NaN	NaN
TCGA-24-1551-01	NaN	NaN
TCGA-24-1552-01	NaN	NaN
TCGA-24-1553-01	NaN	NaN
TCGA-24-1555-01	NaN	X2878_splice,P2608Qfs*40
TCGA-24-1556-01	NaN	NaN
TCGA-24-1557-01	NaN	NaN
TCGA-24-1558-01	NaN	NaN
TCGA-24-1560-01	NaN	NaN
TCGA-24-1562-01	NaN	K3326*
TCGA-24-1563-01	NaN	NaN
TCGA-24-1564-01	NaN	NaN
TCGA-24-1565-01	NaN	NaN
TCGA-24-1567-01	NaN	NaN

TCGA-24-1603-01	NaN	NaN
TCGA-24-1604-01	NaN	NaN
TCGA-24-1614-01	NaN	NaN
TCGA-24-1616-01	NaN	NaN
TCGA-24-2019-01	NaN	NaN
TCGA-24-2024-01	NaN	Y1710*
TCGA-24-2030-01	NaN	NaN
TCGA-24-2035-01	G1710Efs*4	NaN
TCGA-24-2038-01	NaN	NaN
TCGA-24-2254-01	NaN	NaN
TCGA-24-2260-01	NaN	NaN
TCGA-24-2261-01	NaN	NaN
TCGA-24-2262-01	NaN	NaN
TCGA-24-2267-01	NaN	NaN
TCGA-24-2271-01	NaN	NaN
TCGA-24-2280-01	NaN	S1982Rfs*22
TCGA-24-2281-01	NaN	NaN
TCGA-24-2288-01	NaN	V220Ifs*4
TCGA-24-2289-01	NaN	NaN
TCGA-24-2290-01	NaN	NaN
TCGA-24-2293-01	NaN	R2520*
TCGA-24-2298-01	Q1395Lfs*11	NaN
TCGA-25-1313-01	NaN	NaN
TCGA-25-1315-01	NaN	NaN
TCGA-25-1316-01	NaN	NaN
TCGA-25-1317-01	NaN	NaN
TCGA-25-1318-01	NaN	L1491Qfs*12
TCGA-25-1319-01	NaN	NaN
TCGA-25-1320-01	NaN	NaN
TCGA-25-1321-01	NaN	NaN
TCGA-25-1322-01	NaN	NaN
TCGA-25-1324-01	NaN	NaN
TCGA-25-1326-01	NaN	NaN
TCGA-25-1328-01	NaN	NaN
TCGA-25-1329-01	NaN	NaN
TCGA-25-1623-01	NaN	NaN
TCGA-25-1625-01	E116*	NaN
TCGA-25-1626-01	NaN	NaN
TCGA-25-1627-01	NaN	NaN
TCGA-25-1628-01	NaN	NaN
TCGA-25-1630-01	A521Qfs*11	NaN
TCGA-25-1631-01	NaN	NaN

TCGA-25-1632-01	S1217Rfs*21	NaN
TCGA-25-1633-01	NaN	NaN
TCGA-25-1634-01	NaN	X2878_splice
TCGA-25-1635-01	NaN	NaN
TCGA-25-2042-01	NaN	NaN
TCGA-25-2391-01	NaN	NaN
TCGA-25-2392-01	E1346Kfs*20	NaN
TCGA-25-2393-01	NaN	NaN
TCGA-25-2396-01	NaN	NaN
TCGA-25-2398-01	NaN	NaN
TCGA-25-2399-01	NaN	NaN
TCGA-25-2400-01	NaN	NaN
TCGA-25-2401-01	Q1756Pfs*74	NaN
TCGA-25-2404-01	NaN	K343Nfs*6
TCGA-25-2408-01	NaN	NaN
TCGA-25-2409-01	NaN	NaN
TCGA-29-2427-01	L431*	NaN
TCGA-30-1853-01	NaN	NaN
TCGA-30-1862-01	NaN	NaN
TCGA-30-1891-01	NaN	NaN
TCGA-31-1950-01	NaN	NaN
TCGA-31-1953-01	NaN	NaN
TCGA-31-1959-01	NaN	NaN
TCGA-36-1568-01	NaN	NaN
TCGA-36-1569-01	NaN	NaN
TCGA-36-1570-01	NaN	NaN
TCGA-36-1571-01	NaN	NaN
TCGA-36-1574-01	NaN	NaN
TCGA-36-1575-01	NaN	NaN
TCGA-36-1576-01	NaN	NaN
TCGA-36-1577-01	NaN	NaN
TCGA-36-1578-01	NaN	NaN
TCGA-36-1580-01	NaN	NaN
TCGA-57-1582-01	R1726Kfs*3	NaN
TCGA-57-1583-01	NaN	NaN
TCGA-57-1584-01	NaN	N1784Hfs*2
TCGA-57-1993-01	NaN	NaN
TCGA-59-2348-01	E797*	NaN
TCGA-59-2350-01	NaN	NaN
TCGA-59-2351-01	NaN	S1982Rfs*22
TCGA-59-2352-01	NaN	NaN
TCGA-59-2354-01	NaN	NaN

TCGA-59-2355-01	NaN	NaN
TCGA-59-2363-01	NaN	NaN
TCGA-61-1728-01	NaN	NaN
TCGA-61-1736-01	NaN	NaN
TCGA-61-1919-01	NaN	NaN
TCGA-61-1995-01	NaN	NaN
TCGA-61-1998-01	NaN	NaN
TCGA-61-2000-01	NaN	NaN
TCGA-61-2002-01	NaN	NaN
TCGA-61-2003-01	NaN	NaN
TCGA-61-2008-01	W1815*	NaN
TCGA-61-2009-01	NaN	NaN
TCGA-61-2012-01	NaN	NaN
TCGA-61-2016-01	NaN	NaN
TCGA-61-2088-01	NaN	NaN
TCGA-61-2092-01	NaN	NaN
TCGA-61-2094-01	NaN	NaN
TCGA-61-2095-01	NaN	NaN
TCGA-61-2097-01	NaN	NaN
TCGA-61-2101-01	NaN	NaN
TCGA-61-2102-01	NaN	NaN
TCGA-61-2104-01	NaN	NaN
TCGA-61-2109-01	K654Sfs*47	NaN
TCGA-61-2110-01	NaN	NaN
TCGA-61-2111-01	NaN	NaN
TCGA-61-2113-01	NaN	NaN
